# Intestinal microbiome dysbiosis in alcohol-dependent patients and its effect on rat behaviors

**DOI:** 10.1128/mbio.02392-23

**Published:** 2023-11-14

**Authors:** Chuansheng Wang, Junli Yan, Keda Du, Shuai Liu, Jiali Wang, Qi Wang, Huajie Zhao, Min Li, Dong Yan, Ruiling Zhang, Fan Yang

**Affiliations:** 1 The Second Affiliated Hospital of Xinxiang Medical University, Henan Key Laboratory of Biological Psychiatry, Xinxiang Medical University, Xinxiang, China; 2 Department of Pathogeny, School of Basic Medical Science, Xinxiang Medical University, Xinxiang, China; Dartmouth College, Hanover, New Hampshire, USA

**Keywords:** alcohol dependence, gut microbiota, gut mycobiota, fecal microbial transfer, cholecystokinin

## Abstract

Alcohol dependence (AD) is a worldwide epidemic of psychiatric disorders. Gut microbiota dysbiosis may be involved in the development of AD. Recently, the number of studies on the relationship between gut microbiota and alcohol is increasing. However, most previous studies were focused on rodent models and gut microbiota, and the data about the alteration of the gut fungi in AD patients were scarce. Convincing evidence of the gut microbiota’s role in the development of AD is lacking. Here, we investigated the characteristics of gut microbiota (bacterial) and mycobiota (fungal) dysbioses in AD patients and assessed the role of the gut microbiome on the behaviors of rats by fecal microbiota transfer, trying to illuminate the possible microbiota mechanism in AD development. We found that AD patients developed gut microbiota dysbiosis, displayed by the fact that the bacterial genus of *Ruminococcaceae* significantly decreased and that of *Lachnospiraceae* increased and the fungal genus of *Saccharomyces* and *Kurtzmaniella* obviously increased and that of *Candida* decreased. Alcohol consumption disturbs the gut equilibrium between bacteria and fungi. The fungal-to-bacterial species ratio was significantly decreased, and the bacterial-fungal trans-kingdom interactions were reduced. By transplanting the gut microbiota from AD patients to rats, we confirmed that the microbiota from AD patients induced the behavioral alterations associated with alcohol dependence in rats, including increased anxiety- and depression-like behaviors, reduced exploratory and recognition memory, and higher alcohol preference. Meanwhile, the microbiota from the AD patient donor upregulated the expression levels of cholecystokinin receptors in the frontal lobe, hippocampus, and cortex in rats, which might be involved in the development of alcohol dependence. Our results demonstrate that AD patients developed gut bacterial and fungal microbiota dysbioses and the gut microbiota may be involved in the development of alcohol addiction by regulating the endogenous cholecystokinin and related receptors’ expression. Our data open new avenues for adjuvant treating AD patients via administrating the intestinal microbiota.

**IMPORTANCE:**

Intestinal microbiome dysbiosis is associated with psychiatric disease through the “microbiota-gut-brain” axis. Here, we revealed that there was obvious intestinal microbiome (including bacterial and fungal) dysbiosis in alcohol-dependent patients. Alcohol consumption seriously disturbs the gut equilibrium between bacteria and fungi, reduces the interactions among bacterial-fungal trans-kingdom, and increases intestinal permeability. Gut microbiota should be considered as a whole to study the development of alcohol dependence. The gut microbiome of alcohol-dependent patients increased the anxiety- and depression-like behavior in rats. The gut microbiota dysbiosis may promote the development of alcohol dependence by regulating the endogenous cholecystokinin (CCK) and related receptors. Hence, regulating the balance of gut microbiota and the endogenous CCK may be a potential strategy for reducing the risk of relapse in alcohol addiction patients.

## INTRODUCTION

Alcohol dependence (AD), also called alcohol use disorder (AUD), is a common mental disorder that is pervasive worldwide. Alcohol abuse is the third largest risk factor for global morbidity and disability (1). According to the World Health Organization (WHO) 2018 report, 4.9% of the adult population in the world suffers from AD, and 13.5% of all deaths of younger age (from ages 20 to 39) is attributed to alcohol abuse (2, 3). Among the Chinese, a meta-analysis based on relevant documents before January 2014 shows that the current and lifetime prevalence rates of AD were 2.2% and 3.7%, respectively, which are an approach to those of Western countries (4). Especially, with the coronavirus disease 2019 (COVID-19) pandemic, the incidence rate of AD has been higher in comparison to those of previous decades (5). AD not only damages physical and mental health but also brings a heavy burden for patients, families, and society (6, 7), which has brought a pressing and worldwide public health issue. Hence, understanding the pathogenesis of AD development is crucial to preventing and treating this disorder.

In recent years, research based on “the gut-brain axis” theory indicated that gut microbiota dysbiosis might be involved in the development of psychiatric disorders including alcohol craving and dependence (8–10). It is well known that gut microbial composition and activity can be modified by many factors, such as antibiotic use, diet, and other environmental factors (11–13). It has been proved that alcohol consumption could change the microbial composition of the gut and induce gut dysbiosis in rodent and non-human primate studies, as well as patients with alcohol use disorder (14–17). Meanwhile, some research also observed that the changes in the gut microbiome were associated with depression- and anxiety-like behavioral alterations in rodent models of alcoholism (18, 19), which suggested that there was a certain relationship between intestinal microbiota and the development of AD. At present, the number of studies on the relationship between gut microbiota and AUD is increasing. However, we found that there were some flaws in the present research. Firstly, most previous studies focused on rodent models but not on human patients, just as Hillemacher et al. described in a review which stated that clinical studies of gut microbiota in alcohol-dependent humans had hitherto been sparse (20). Secondly, the gut microbiome is made up of bacteria, fungi, and viruses. Existing research on the gut microbiome of alcohol-related disease has almost exclusively focused on gut bacteria, with the literature regarding alterations of gut fungal and viral ecology being scarce. Thirdly, previous studies have independently focused on describing the effects of alcohol consumption on intestinal microbiota and intestinal barrier in animal models and patients. Few researchers have systematically investigated the further effects of gut microbiome dysbiosis induced by alcohol intake on behaviors, and the possible pathogenesis of gut microbiota dysbiosis contributing to alcohol-dependent development also remains unclear.

Our previous studies have indicated that AD rats showed gut microbiota dysbiosis and gut permeability disorder (21, 22). However, due to the species differences, the alteration traits of the gut microbiota composition induced by alcohol in animals are not completely identical to those in humans. Hence, in the present study, we select clinical AD patients to investigate the characteristics of gut microbiota (bacterial) and mycobiota (fungal) dysbioses. Furthermore, we investigate the effect of AD patient gut microbiome on the behaviors of rats with fecal microbiome transplantation technology and try to explore the potential role and mechanism of gut microbiome dysbiosis in promoting AD development.

## RESULTS

### Participants’ demographics

Henan Mental Hospital is the largest specialized psychiatric hospital in Henan province, China. It is located in Xinxiang City (in 35°18′ North and 113°55′ East longitude) (Fig. S1), China. Approximately, 3,500 AD patients visit the addiction department of the hospital and 800 AD patients need medical treatment in the hospital each year. We recruited 264 alcohol-dependent patients and 45 healthy controls from January to June 2021. After screening by inclusion and exclusion criteria, a total of 34 AD patients and 19 healthy control subjects were recruited for this study. All subjects in this study were male because few patients with AD treated at hospitals in the area were female. The demographic and clinical characteristics of the involved subjects are presented in Table 1, and we found that the two groups were similar regarding age and body mass index (BMI), which indicated that the subjects in each group were comparable. All patients were evaluated for a diagnosis of AD by a psychiatrist according to the Alcohol Use Disorder Identification Test (AUDIT). Meanwhile, the levels of depression, anxiety, alcohol withdrawal, and craving of all patients have been assessed using the Hamilton Depression Scale (HAMD), Hamilton Anxiety Scale (HAMA), Clinical Institute Withdrawal Assessment (CIWA), and Penn-Alcohol Craving Scale Day (PACS) criteria, respectively. The results showed that AD patients were obviously different from the control subjects (Table 1), which indicated that all the 34 AD participants met the criteria for AD.

**TABLE 1:** Baseline characteristics of study participants^*a*^

Variable	Alcohol dependent	Control group	*P*-value
Number of participants	34	19	
Gender	Male	Male	
Age (years)	39.24 ± 1.72	39.58 ± 2.76	0.649
BMI (kg/m^2^)^*b*^	24.6 ± 3.4	25.6 ± 2.4	0.437
Alcohol intake (g/day)	489.42 ± 29.91	8.68 ± 2.22	0.001
Duration of drinking habit (years)	15.82 ± 9.04	NA	
AUDIT score	33.59 ± 0.39	1.47 ± 0.50	<0.001
CIWA score	14.91 ± 1.07	0	<0.001
HAMA	9.44 ± 1.08	1.16 ± 0.34	<0.001
HAMD	9.12 ± 0.86	2.32 ± 0.38	<0.001
PACS	16.09 ± 1.079	0	<0.001
MMSE score	27.21 ± 0.48	29.58 ± 0.22	<0.001

^*a*^ Data are means ± SEM. *P*-values were calculated using a *t*-test or Mann-Whitney’s test.

^*b*^ BMI, body mass index; AUDIT, Alcohol Use Disorder Identification Test; CIWA, Clinical Institute Withdrawal Assessment; HAMA, Hamilton Anxiety Scale; HAMD, Hamilton Depression Scale; PACS, Penn-Alcohol Craving Scale Day; MMSE, Mini-Mental State Examination.

### The changes in serological biochemical parameters in AD patients

**Fig 1:**
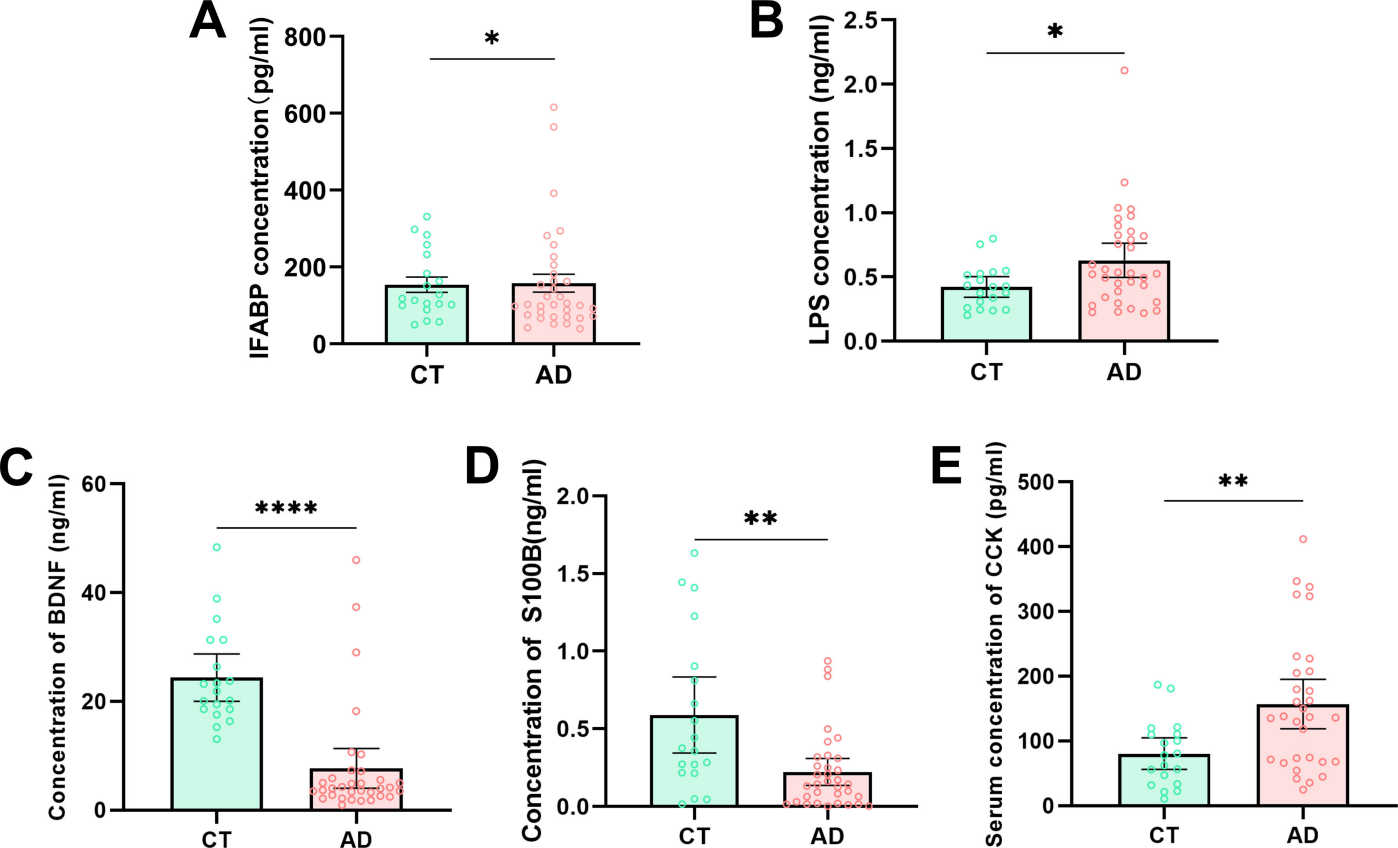
The comparison of serological parameters between AD patients and health control subjects. (**A**) Intestinal fatty acid binding protein (i-FABP). (**B**) Lipopolysaccharides (LPS). (**C**) Brain-derived neurotrophic factor (BDNF). (**D**) S100 calcium-binding protein B. (**E**) Cholecystokinin (CCK). AD, alcohol-dependent group, *n* = 34; CT, control group, *n* = 19. *P < 0.05, **P < 0.01, and ***P < 0.001.

To assess the effect of long-term alcohol ingestion on gut mucosal damage and permeability, the intestinal fatty acid binding protein (i-FABP, a marker of enterocyte death) and lipopolysaccharides (LPS, an endotoxin, as a marker of gut permeability) in serum were investigated with enzyme-linked immunosorbent assay (ELISA) technology. The results showed that the levels of i-FABP and LPS were significantly higher in AD patients than in the healthy control group (CT) (Fig. 1A and B), which indicated that long-term alcohol consumption damages the enterocyte and increases gut permeability and microbial translocation. The brain-derived neurotrophic factor (BDNF) is a neurotrophic neuropeptide, which modulates the development of addictive behavior (23). Our data showed that BDNF serum levels were obviously decreased in AD patients compared with the control group (*P* < 0.001, Fig. 1C). S100B is a calcium-binding protein that has been proposed as a marker of astrocyte activation and brain dysfunction (24). We detected the serum levels of S100B protein in the present study, and the results showed the levels of S100B were significantly lower in the AD group than in the control group (*P* < 0.01, Fig. 1D). Besides, we also investigated the serum levels of cholecystokinin (CCK), an important gastrointestinal peptide hormone. We found that the levels of CCK were dramatically increased in AD patients compared with control health people (*P* < 0.01, Fig. 1E). Together, alcohol consumption increased gut permeability and altered some serological biochemical parameters.

### The gut microbiota altered in AD patients

**Fig 2:**
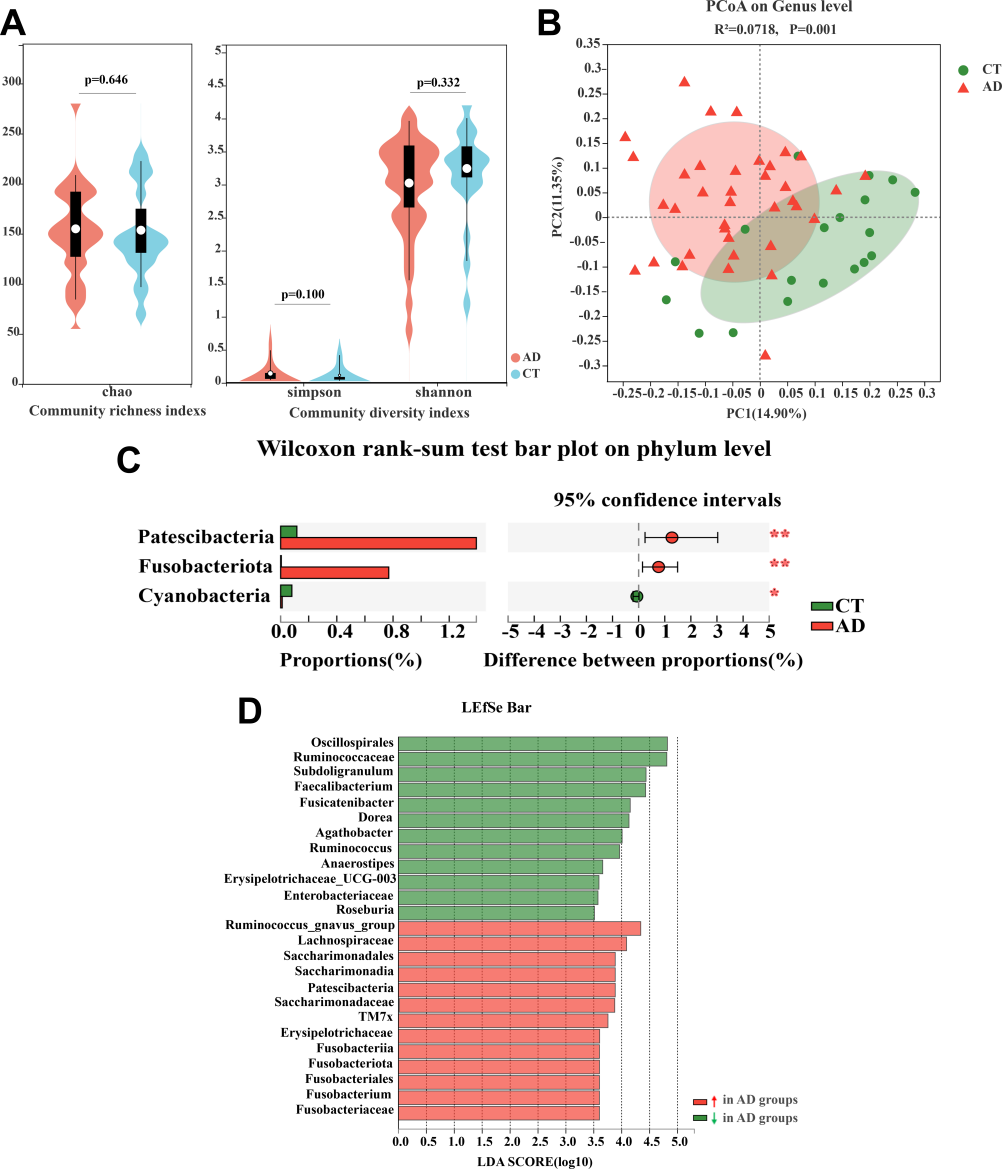
Alcohol-dependent patients show significant alterations in microbiota composition profiles. (**A**) Alpha diversity of gut microbiota in AD and CT subjects. (**B**) PCoA score plots of amplicon sequence variants (ASVs) based on Unweighted UniFrac values between AD and CT groups. (**C**) The relative abundance difference of gut microbiota at the phylum level between the AD and CT groups with the Wilcoxon rank-sum test. (**D**) The LEfSe analysis of taxonomic distribution in the different groups (LDA score > 3.5). AD, alcohol-dependent group; CT, control group. **P < 0.01.

The richness index (Chao) and diversity indexes (Shannon and Simpson) were used to assess the effect of alcohol consumption on human gut microbiota. The results showed that there were no significant differences between the AD patients and the control health subjects (Fig. 2A), which indicated that alcohol consumption did not affect the gut microbiota diversity. The principal coordinate analysis (PCoA) based on Unweighted UniFrac values and permutational multivariate analysis of variance (PERMANOVA) at the genus level revealed that the intestinal microbial community structure had a significant difference between the AD patients and the control health subjects (Fig. 2B, *R*^2^ = 0.0718; *P* = 0.001). To further assess which bacteria are predominantly affected by alcohol overconsumption, we focused on the analyses of bacterial taxa relative abundance at the phylum and genus levels. The results showed that, at the phylum level, four phyla including *Firmicutes*, *Proteobacteria*, *Bacteroidetes*, and *Actinobacteria* were dominant and there was no significant difference between the two groups of subjects (Table S1). However, *Patescibacteria* and *Fusobacteriota* had an obviously higher relative abundance in AD patients than in healthy subjects (Fig. 2C; Table S1). Then, we used linear discriminant analysis effect size (LEfSe) to assess the difference in abundance at the genus level. We found that *Saccharimonadaceae*, *Lachnospiraceae*, and *Fusobacterium* increased remarkably in AD patients (Fig. 2D). Furthermore, we also observed that three genera in the family *Ruminococcaceae* decreased in AD patients (Fig. 2D; Table S2). These genera belong to the class *Clostridia* of *Firmicutes* phylum. Of note, the genera of *Roseburia* and *Erysipelotrichaceae_UCG-003* decreased in the AD patient group vs the control group (*P* = 0.004, 0.037), which also showed the same results as our previous study on alcohol-dependent rats’ model. A cladogram is performed to illustrate the relationships between different taxa, and the results show that the effect of alcohol on gut microbiota mainly focuses on *Firmicutes*, *Patescibacteria*, and *Fusobacteriota* (Fig. S2). Altogether, these results indicate that alcohol consumption changes the gut microbial composition pattern.

### Altered gut mycobiota profile in AD patients

**Fig 3:**
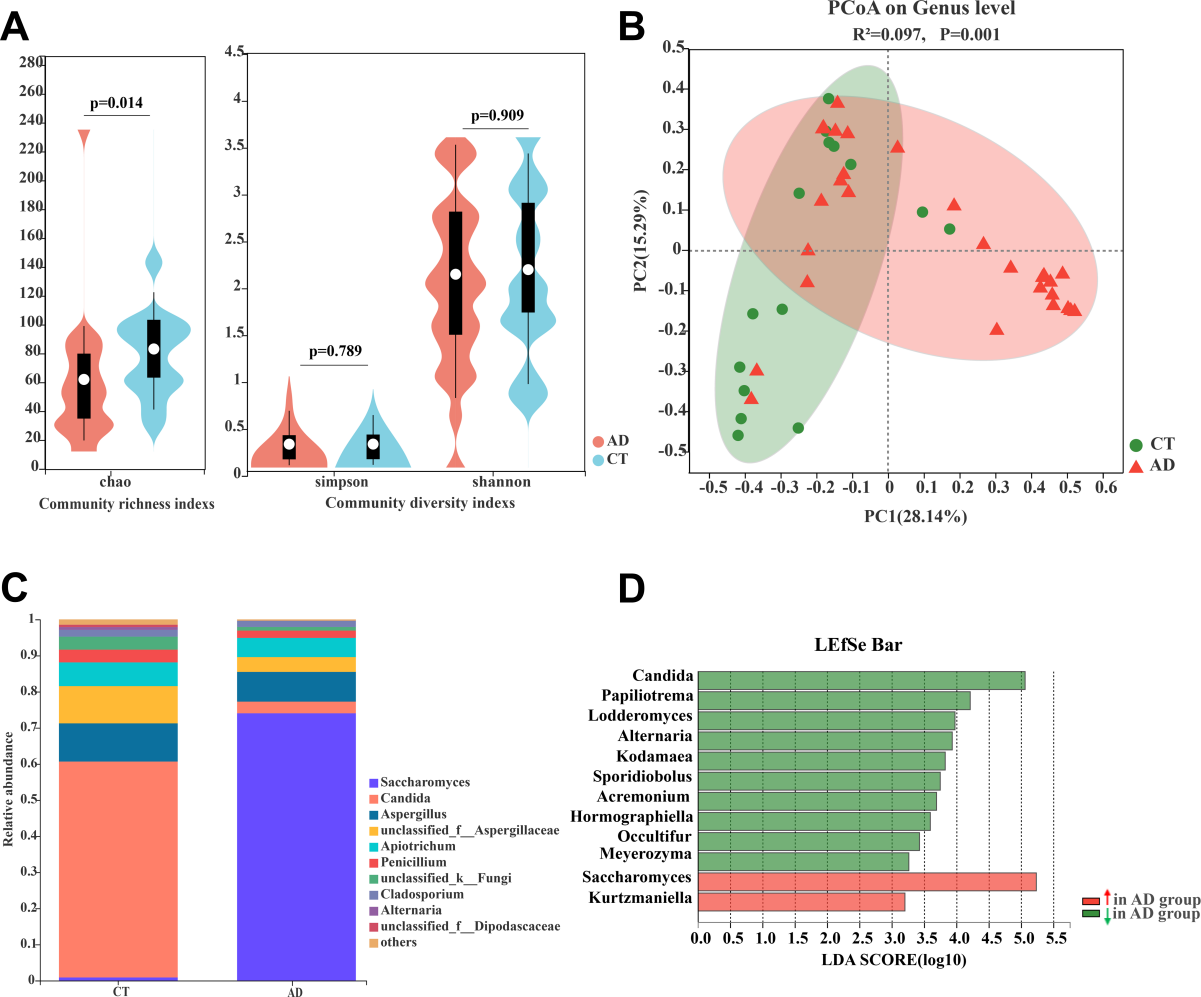
Alcohol-dependent patients show significant alterations in gut fungal composition profiles. (**A**) Alpha diversity of gut fungi in AD and CT subjects. (**B**) PCoA score plots of ASVs based on unweighted UniFrac values between AD and CT groups. (**C**) The relative abundance of gut fungal composition at the genus level. (**D**) The LEfSe analysis of taxonomic distribution in the different groups (LDA score > 3.0). AD, alcohol-dependent group; CT, control group.

We assessed the changes induced in the gut mycobiome from alcohol overconsumption by Internal Transcribed Spacer 1 (ITS1) sequencing. ITS1 sequences were clustered into amplicon sequence variants and taxonomically annotated using Classify-sklearn (Naive Bayes) and RDP classifier on the Majorbio Cloud platform (https://cloud.majorbio.com/). The alpha diversity analyses showed that the Chao index in AD patients was significantly lower than that in the control subjects (*P* = 0.014, Fig. 3A). However, similar to the bacterial gut microbiota, the Shannon and Simpson indexes showed no obvious differences between AD and CT groups (Fig. 3A). This indicates alcohol overconsumption can deplete the gut fungi. Based on the Bray-Curtis dissimilarities, the PCoA and PERMANOVA analysis showed that the gut mycobiota of AD was significantly separated from the CT group (*R*^2^ = 0.097; *P* = 0.001; Fig. 3B). Subsequently, we investigated the differences of fungal relative abundance at phylum and genus levels. A total nine fungal phyla were found in both groups, and the *Ascomycota* (73.84% AD, 71.25% CT) and *Basidiomycota* (24.2% AD, 27.48% CT) phyla were the dominant taxa in both groups. The fungal relative abundance at the phyla level showed no significant differences between AD and CT groups (*P* > 0.05, Table S3). Genus-level analysis showed that *Saccharomyces* and *Candida* were the most dominant genus. Using the Wilcoxon rank-sum test analysis, it is shown that the relative abundance of *Saccharomyces* was significantly enriched in the AD group but depleted in the CT group (*P* < 0.001, Fig. 3C; Table S4). However, the abundance of genera *Candida* obviously decreased in the AD group vs CT group (*P* = 0.032, Fig. 3C; Table S4). Furthermore, with LEfSe analysis, we observed that the genus *Kurtzmaniella* also increased remarkably in the AD group, and 10 genera including *Papiliotrema*, *Lodderomyces*, *Alternaria*, and *Kodamaea* decreased in the AD group (Fig. 3D). All these data reveal that alcohol overconsumption obviously alters the gut fungal mycobiota diversity and composition profile.

### AD patients altered the bacterial-fungal trans-kingdom network construction

**Fig 4:**
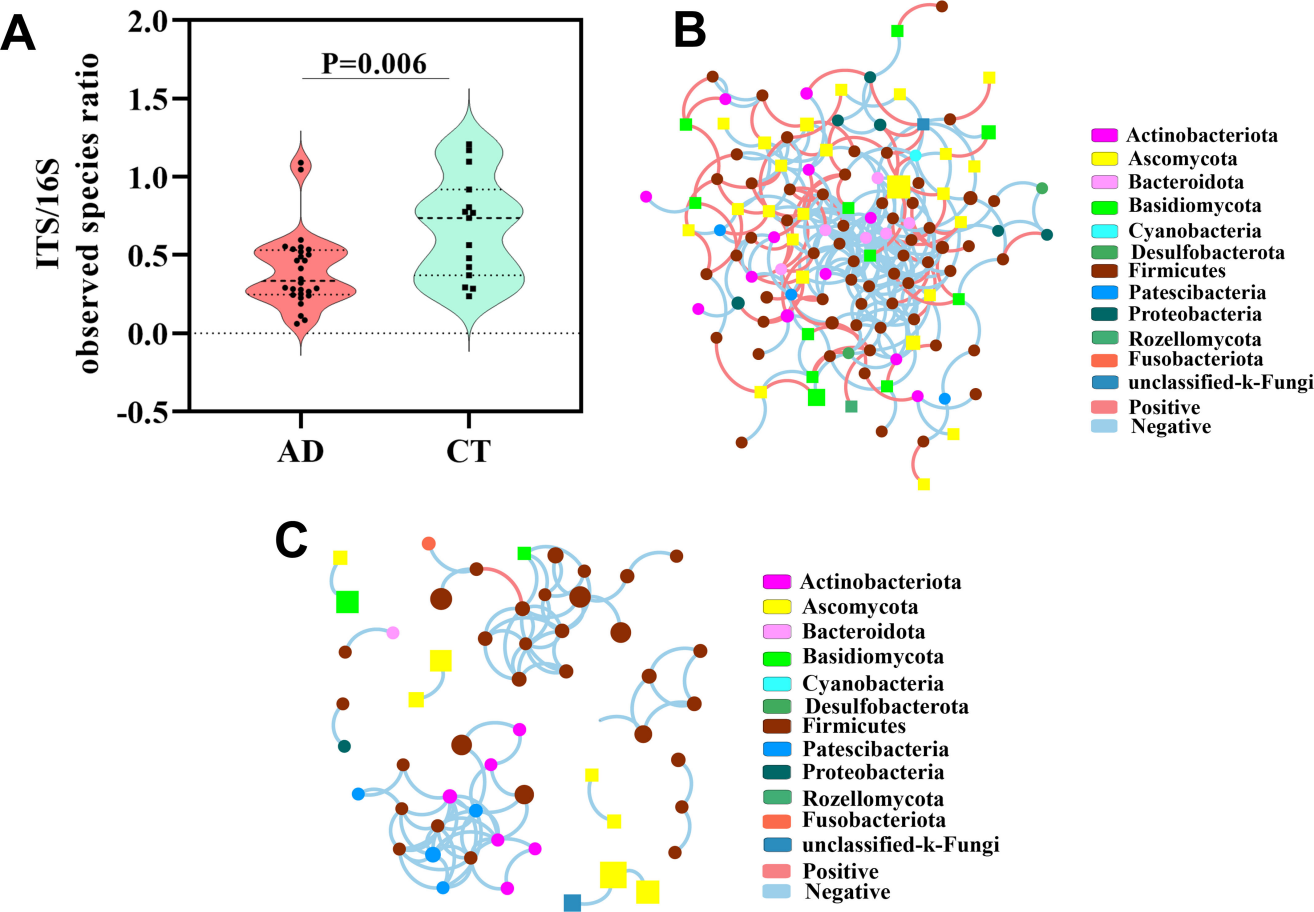
Fungal-bacterial equilibration analyses in two groups. (**A**) The ITS/16S diversity ratio (Wilcoxon rank sum test). The trans-kingdom abundance correlation networks in the CT group (**B**) and AD group (**C**) at the genus level. Each node represents a genus, and its color represents the phylum to which it belongs. Squares represent fungi and circles represent bacteria. Node size represents the mean abundance of each genus. Edges indicate the magnitude of correlations, positive in red and negative in blue. AD, alcohol-dependent group; CT, control group.

To explore the effect of alcohol intake on the gut bacterial and fungal diversity balance, we analyzed the fungal-to-bacterial species ratio based on observed Sobs indexes of the ITS/16S. The results showed that the ITS/16S ratio of the AD group was significantly decreased compared with the CT group (*P* = 0.006, Fig. 4A), which indicated that alcohol overconsumption disturbed the gut equilibrium between bacteria and fungi. Next, we performed a trans-kingdom network analysis between bacteria and fungi to assess the interplay at the genus level. The results showed that the microbiome network (including bacteria and fungi) in AD patients was obviously different from that in the CT group (Fig. 4B through C). In the healthy control subject group, we found that the bacteria and fungi were related to each other, gathering in a cluster and forming a more complex network (Fig. 4B), in which a total of 126 nodes were found at the genus level and the ratio of fungi and bacteria was 0.42 (37/89) (Table 2). However, the complexity of the network in the AD group was notably lower and the relationship between bacteria and fungi was also weaker (Fig. 4C). A total 55 nodes gathered to clusters in this network (Table 2). Meanwhile, we also found the dyads, triads, and quintuplets in the network of the AD group had no connection with the major network (Fig. 4C). The network centralization and density of fungi were remarkably reduced compared with CT group, and the ratio of fungi and bacteria was 0.22 (10/45) (Table 2). All these results suggested that alcohol consumption obviously altered the bacterial-fungal trans-kingdom network, reduced the interactions between bacteria and fungi, disturbed the entire ecosystem balance of the gut microbiome, and resulted in gut microbiome dysbiosis.

**TABLE 2:** The parameters of the trans-kingdom networks of each group

Parameters	Control group	Alcohol-dependent group
Nodes (n) (fungi/bacteria)	126 (37/89)	55 (10/45)
Edges (n) (positive/negative)	273 (199/74)	86 (85/1)
Relative connectedness	2.17	1.56
Clusters (n)	1	10

### The metabolic profile is altered in AD patients

**Fig 5:**
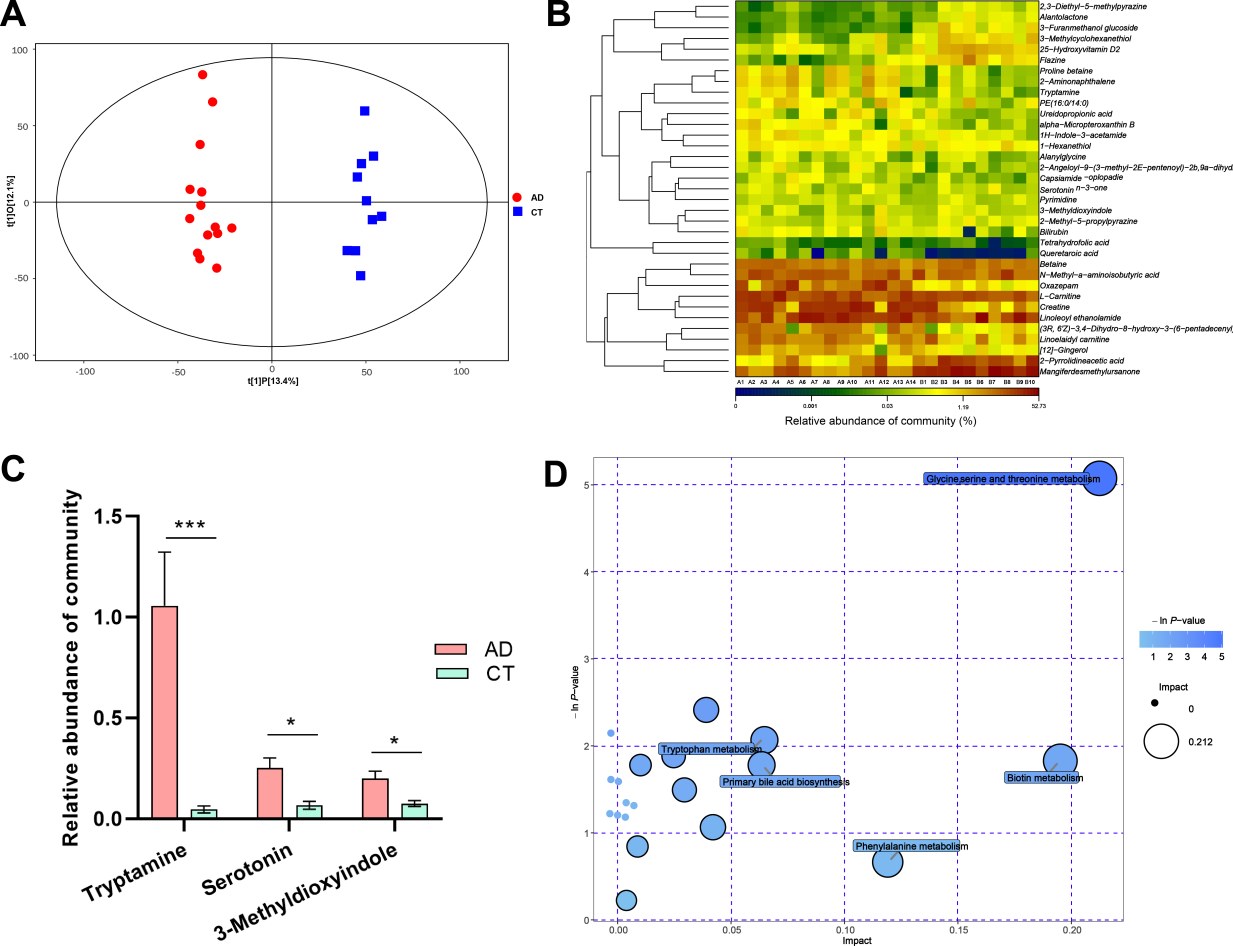
Untargeted metabolomics of the fecal metabolomes in AD vs CT group subjects. (**A**) The OPLS-DA score plot of metabolites in feces from different groups, (*R*^2^*Y* = 0.85, *Q*^2^ = 0.79). (**B**) The heatmap of the significantly different metabolites between AD and CT groups, FDR < 0.05. (**C**) The relative abundance of a community of neurotransmitters or derivatives in different groups (Wilcoxon rank sum test). (**D**) The topology analysis of KEGG metabolic pathway. AD, alcohol-dependent group; CT, control group. *P < 0.05, **P < 0.01, and ***P < 0.001.

An untargeted metabolomic analysis using liquid chromatography-mass spectrometry (LC-MS) was performed to assess the metabolite profiles of AD patients versus control healthy subjects. The principal component analysis (PCA) results showed that the QC (quality control) samples were tightly clustered in the center on PCA scatter plots (Fig. S3), which indicated that our analyses were reliable. The metabolites of the AD group were obviously distinguished from the CT group on the PCA score plot (Fig. S3). Next, we constructed an orthogonal partial least squares discriminant analysis (OPLS-DA) model to maximize the identification of differential metabolites between the AD and CT groups. The results revealed that the metabolites of alcohol-dependent patients and the control group were clearly dispersed in two different regions (Fig. 5A). The permutation test showed that the OPLS-DA model was not overfitted and was with good predictive power (*R*^2^*Y* = 0.85, *Q*^2^ = 0.79, Fig. S4). In this study, a total of 180 metabolites were identified in both groups. Using the parameters of variable importance in projection (VIP) > 1.5 and *P* < 0.05, we further screened the differential metabolites between the two groups and found a total of 90 metabolite biomarkers altered obviously in AD patients (Table S5). Among these compounds, the levels of 53 metabolites were upregulated and 37 metabolites were downregulated in the AD patients (Fig. S5; Table S5). We further performed a calibration with a falsely discovery rate (FDR) and selected *q* < 0.05 as a screening criterion, and a total of 35 metabolite biomarkers were significantly changed. The heatmap of differential metabolites revealed that compared with the healthy control group, the levels of 27 metabolites including creatine, alanylglycine, bilirubin, L-carnitine, and tetrahydrofolic acid were significantly upregulated in AD patients. Conversely, the levels of flazine, 25-Hydroxyvitamin D2, mangiferdesmethylursanone, other eight metabolites were downregulated in the AD patients (Fig. 5B; Table S5). Interestingly, we found the levels of indoles and derivatives including tryptamine, serotonin, and 3-methyldioxyindole were significantly increased in the AD group vs CT group (Fig. 5C). These metabolites are neurotransmitters or their derivatives, which were involved in the regulation of addictive behaviors (25, 26). Next, we carried out the metabolic pathways based on these differential metabolite biomarkers and found that 20 key metabolic pathways might be involved in alcohol-dependent development (Table S6). Especially, glycine, serine, and threonine metabolism pathways were significantly different between the AD group and the CT group (*P* = 0.006, impact value = 0.212, Fig. 5D; Table S6). Taken together, these data reveal that alcohol consumption significantly disturbs the fecal metabolic profile and metabolic pathways in AD patients.

### Potential correlations between the gut microbiota and fecal metabolites

**Fig 6:**
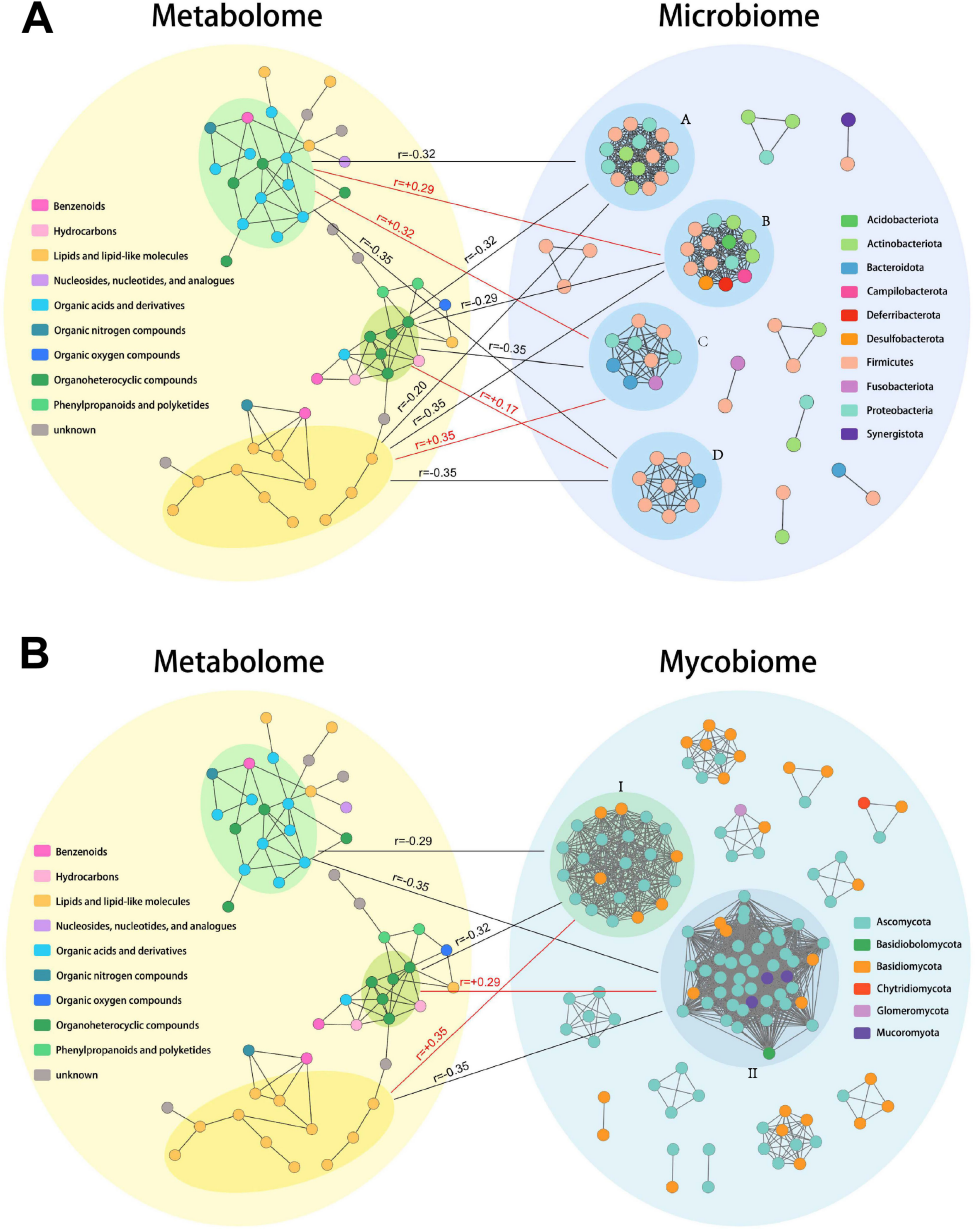
The results of association analysis. (**A**) Network analysis between the microbiome (bacteria) and metabolome. (**B**) Network analysis between the mycobiome (fungi) and metabolome. The *R*-value represents Spearman’s rank correlation coefficient. The associations were assessed by linear regression analysis after adjusting for alcohol consumption. The red line represents a positive correlation and the black line is a negative correlation.

We performed two correlation analyses to investigate the relevance between alterations of gut microbiota and fecal metabolites. Based on the Spearman correlation coefficients, we calculated the interaction correlation between the microbiome and metabolome and performed an interaction network analysis. As Fig. 6A shows, in the microbiota networks, there were four subnetworks (A, B, C, and D). One subnetwork (D) was mainly composed of members of the *Firmicutes* phylum. The other three subnetworks were composed of several phyla including *Firmicutes*, *Proteobacteria*, *Bacteroides*, and *Acidobacteriota*. However, there were three metabolite subnetworks in the metabolome networks, one of which (yellow in Fig. 6) was independent clusters of lipids and lipid-like molecules and the other two (green and blue in Fig. 6) were a mixed group including organic acids and derivatives, benzenoid, and organic heterocyclic compounds. The D subnetwork of the microbiome (mainly including *Firmicutes*) was negatively correlated with lipid molecules (*r* = −0.35) and organic acids (*r* = −0.35) (Fig. 6A). The A subnetwork of the microbiome was negatively correlated with all three metabolite subnetworks. The B subnetwork of the microbiome was negatively correlated with lipids and organic heterocyclic compounds (*r* = −0.35 and *r* = −0.29, respectively) and was positively correlated with organic acids (*r* = 0.29). In the mycobiome networks, there were two main subnetworks; one (I type) was composed of *Ascomycota* and *Basidiomycota* and the other (II type) was composed of *Ascomycota*, *Basidiomycota*, and *Mucoromycota* (Fig. 6B). I type subnetwork of mycobiome was positively correlated with lipid (*r* = 0.35) and negatively related with organic heterocyclic compounds (*r* = −0.32) and organic acids (*r* = 0.29). II type subnetwork of mycobiome was negatively related with lipid (*r* = −0.35) and organic acids (*r* = −0.35) and positively with organic heterocyclic compounds (*r* = 0.29) (Fig. 6B). All these results indicate that the alterations in the gut microbiome are closely related to the changes in fecal metabolites in alcohol-dependent patients.

### Fecal microbial transfer from AD patients’ donors to antibiotics-treated conventional rats

**Fig 7:**
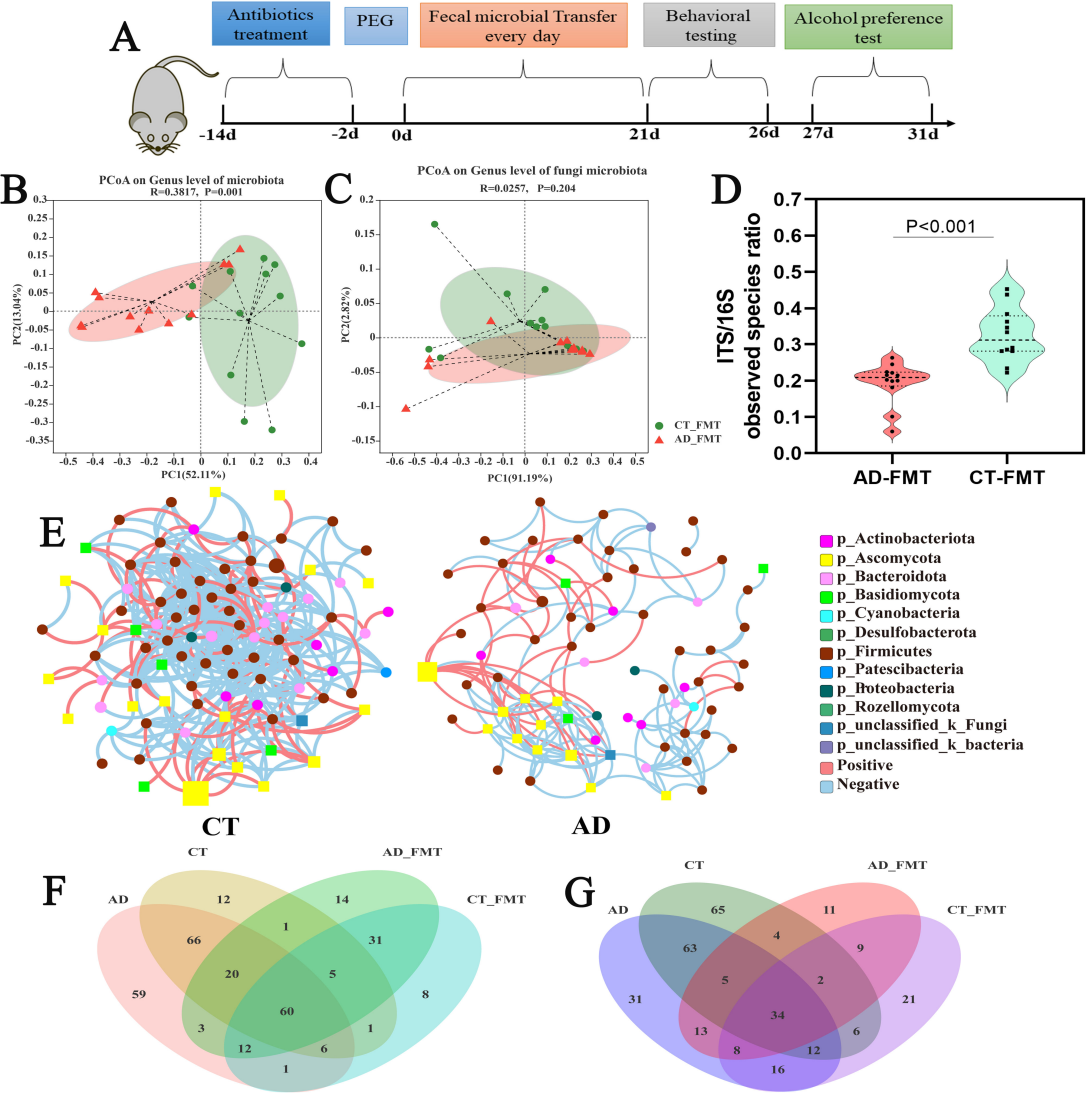
Design of the FMT experiment and analysis of the gut microbiome (bacteria and fungi) alteration of the transplanted rats. (**A**) The experimental procedures of the transplantation of feces from AD patients and healthy control humans to rats. (**B**) The PCoA score plots of bacterial microbiota at the genus level based on Unweighted UniFrac values between AD-FMT and CT-FMT groups. (**C**) The PCoA score plots of fungal mycobiota at the genus level between AD-FMT and CT-FMT groups. (**D**) The observed species rations of fungi and bacteria in FMT rats (Wilcoxon rank sum test). (**E**) The trans-kingdom abundance correlation networks in the CT-FMT group and AD-FMT group at the genus level. (F) The Venn diagram of gut microbiota in different groups at the genus level. (**G**) The Venn diagram of gut fungi in different groups at the genus level. FMT, fecal microbiota transplantation; AD, alcohol dependent group; CT, control group.

The above observations show alcohol consumption induces gut microbiome dysbiosis in AD patients. However, after alcohol withdrawal, the clinical symptoms associated with alcohol dependence will continue, and this disease is very easy to relapse. Hence, we postulate that gut microbiome dysbiosis plays a potential role in promoting and maintaining the development of alcohol dependence. To assess this hypothesis, we performed a fecal microbiome transfer (FMT) model to investigate the effects of gut microbiome dysbiosis on host addiction-associated behaviors and underlying physiological and molecular alterations. The experimental design for the FMT is shown in Fig. 7A. First, we measured the efficacy of depleting the gut microbiota by the antibiotics treatment, and the results showed that the bacterial DNA loading in the rat’s feces was dramatically reduced (>300-fold, Fig. S6) after antibiotic cocktail treating for 12 days. Then, we analyzed the changes in gut bacterial and fungal microbiota between AD-recipient rats and CT-recipient rats. The β-diversity analysis (PCoA) showed a significant separation of gut bacterial microbiota between CT- and AD-recipient samples along the longitudinal axis (*P* = 0.001, Fig. 7B). However, the gut fungal microbiota had no obvious distinction between the two groups (*P* = 0.204, Fig. 7C). To perform a comprehensive analysis of the gut bacterial and fungal diversity balance, we analyzed the fungal-to-bacterial species ratio based on the observed Sobs indexes of the ITS/16S, and the results showed that the ITS/16S ratio was significantly reduced in the AD-FMT group vs CT-FMT group (*P* < 0.001, Fig. 7D). The trans-kingdom network analyses between bacteria and fungi showed, compared with that in the CT-FMT group, the complexity of the microbiome network significantly reduced in AD-FMT group, and the relationship between bacteria and fungi was also decreased (Fig. 7E). These change trends of the gut microbiome in recipient rats were consistent with those in the human donors. To assess the microbiome profile of rats after FMT, we performed the Venn diagram to identify the common or unique community between groups at the genus level. The results showed that 41.85% (95/227) of bacterial genera and 32.97% (60/182) of fungal genera present in the AD inocula were successfully transferred to AD-recipient rats and 42.11% (72/171) of bacteria genera and 28.27% (54/191) of fungal genus in the CT inocula were also successfully transferred to CT-recipient rats, respectively (Fig. 7F and G). Together, these results confirm that the FMT model established by us was successful and the AD-recipient rats present the disorder of gut microbiota ecosystem balance, which is similar to AD patients. Hence, this model is suitable for assessing the effect of gut dysbiosis induced by alcohol intake on rat behavior.

### Rats inoculated with AD microbiota exhibit change in behavior

**Fig 8:**
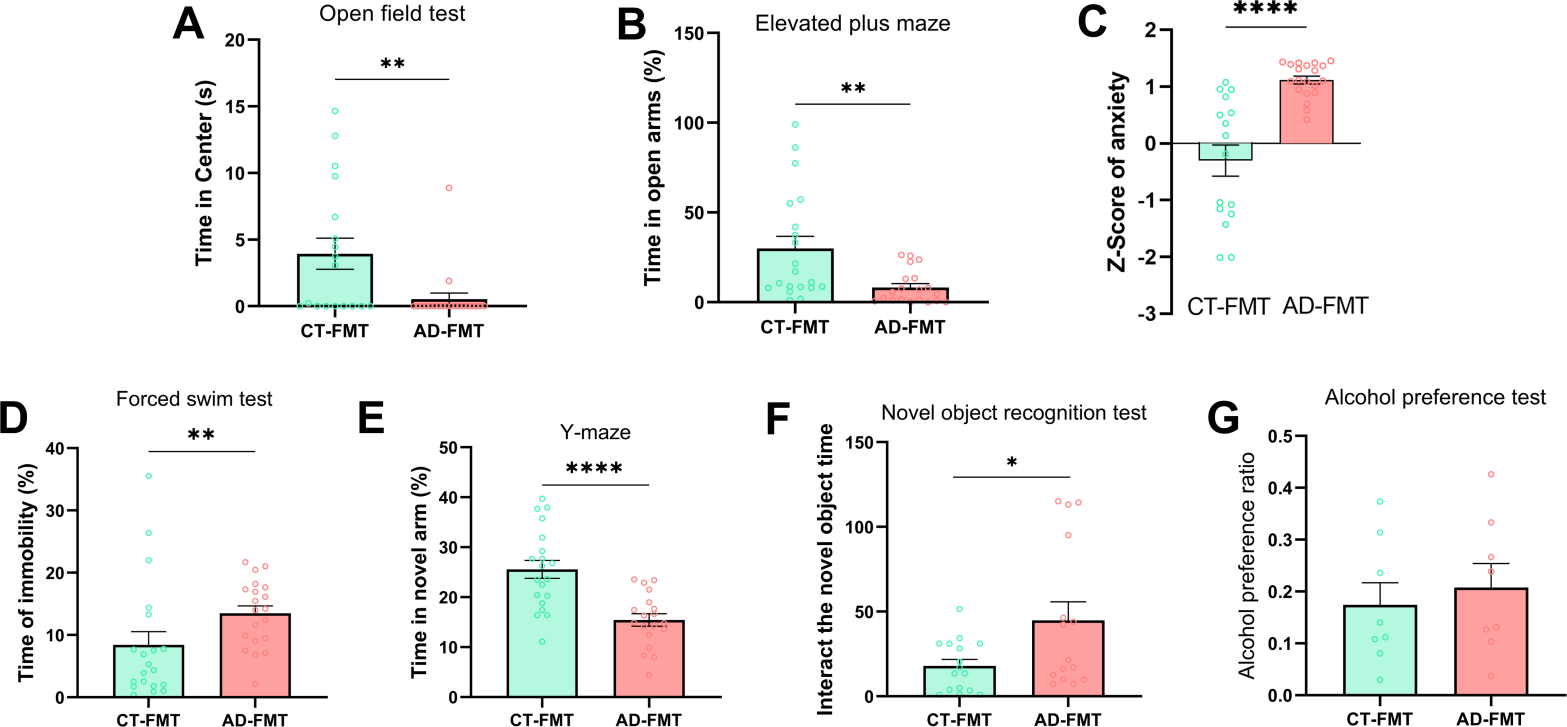
Impact of FMT on rat’s behavior. (**A**) Bar graphs show the time spent in the central zone of the open field test. (**B**) The time spent in the open arms of the EPM. (**C**) Z-score of anxiety, based on EPM and open field test. (**D**) Time spent immobile during FST. (**E**) Time spent in the novel arm during the Y-maze test. (**F**) Time of interaction with the novel object in NORT. (**G**) The alcohol preference ratio of AD-recipient rats vs CT-recipient rats. Data represent mean ± SD, Student’s unpaired *t*-test. *P < 0.05, **P < 0.01, and ***P < 0.001.

To assess the effects of gut microbiota on host addiction-associated behaviors, a series of tests for detecting anxiety-, depression-, and cognition-like behaviors were performed in CT- and AD-recipient rats. First, we measured the anxiety-like behavior by the open field test (OFT) and the elevated plus maze (EPM). The results showed that there were no obvious differences between the two groups in general locomotor activity in these tests (Fig. S7A and B). However, AD-recipient rats spent significantly less time in the center regions of the OFT test (*P* = 0.0021, Fig. 8A) and the open arms of the EPM test (*P* = 0.0015, Fig. 8B) compared with CT-recipient rats, which indicated that AD-recipient rats displayed higher anxiety. Using these behavioral data, the Z-score for anxiety was calculated, which showed consistent differences in anxiety-like behaviors between AD- and CT-recipient rats (Fig. 8C). Then, we assessed the difference between the two groups in depression-like behaviors. Immobility time in the forced swimming test (FST) is a marker for measuring depression. The results showed that AD-recipient rats exhibited significantly more immobility time in FST than CT-recipient rats (*P* = 0.0056, Fig. 8D), which suggested that the Wistar rats developed depression-like behaviors after receiving fecal microbiota from AD patients. Furthermore, we performed the Y-maze and the novel object recognition test (NORT) to investigate the difference in cognition-like behavior after FMT. The results of the Y-maze test showed that AD-recipient rats spent less time in the novel arm compared with the CT-recipient group during the retention phase (*P* < 0.0001, Fig. 8E), which indicates that fecal microbiota from AD patients may impair spatial recognition memory of rats. AD-recipient rats spent more time interacting with the novel objects (*P* = 0.0247) and displayed higher thigmotactic behavior compared with CT group rats (*P* = 0.0352) in NORT (Fig. 8F), indicating AD-recipient rats show less exploratory and recognition memory. The results of the alcohol preference test showed that there was no significant difference in alcohol consumption between both groups, but AD-recipient rats presented a higher alcohol preference tendency vs CT-recipient rats (Fig. 8G). Collectively, the above results showed that the FMT from AD patients to rats is sufficient to induce similar clinical features of alcohol dependence including anxiety, depression, and cognitive impairment, which means gut microbiota dysbiosis plays an important role in promoting the development of alcohol dependence.

### Rats inoculated with the AD microbiota exhibit changes in CCKR in the brain

**Fig 9:**
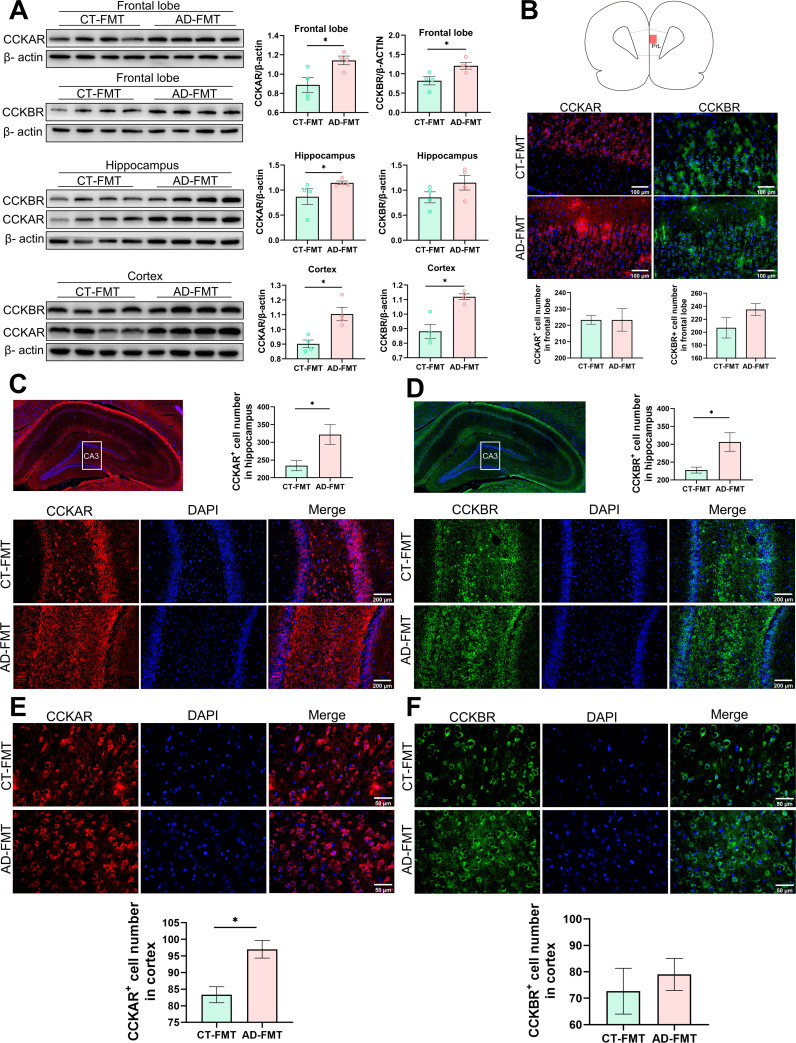
Effect of fecal microbiota transplantation on CCKR expression in the brain. (**A**) Western blotting images and analysis of CCKAR and CCKBR expression in the frontal lobe, hippocampus, and cortex. (**B**) The expression and analysis of CCKAR and CCKBR in the frontal lobe were identified by immunofluorescence. The expression and analysis of CCKAR (**C**) and CCKBR (**D**) in the hippocampus (CA3) were identified by immunofluorescence. The expression and analysis of CCKAR (**E**) and CCKBR (**F**) in the cortex were identified by immunofluorescence. Scale bar: 100 μm in panel B, 200 μm in panels C and D, and 50 μm in panels E and F. Data were represented as mean ± SEM. Significant differences were assessed by the Mann-Whitney test or unpaired *t*-test. *P < 0.05 and **P < 0.01.

The possible molecular mechanisms of the AD-recipient rat’s behavioral changes induced by FMT were investigated in the brain and in peripheral metabolism to link these two distant phenomena. Since the CCK and BDNF concentration changes in peripheral blood had been found in alcohol-dependent patients, we wondered whether these changes would cause the expression alteration of related receptors in the brain to regulate the rat’s behavior. Hence, we collected three brain areas including the frontal lobe, hippocampus, and cortex, to investigate the change trend of CCK receptors (CCKR) and BDNF levels between AD- and CT-recipient rats. Firstly, the western blotting (WB) results showed that the expression of the BDNF level in these three brain areas was not significantly different between the two groups (Fig. S8A). Both subunits A and B of CCK receptors (CCKAR and CCKBR) in the frontal lobe and cortex areas were obviously upregulated in AD-recipient rats (Fig. 9A). In addition, we found that the levels of CCKAR also significantly increased in the hippocampus of AD-recipient rats, whereas CCKBR in this area was not obviously different between two groups (Fig. 9A). Then, we further used the immunofluorescence section techniques to verify the above-observed consequence. The results indicated that the BDNF level of the AD-recipient rats was significantly downregulated in the frontal lobe, hippocampus, and cortex (Fig. S8B to D). CCKBR was not obviously different in the frontal lobe and cortex between the two groups (Fig. 9B and F). However, in the hippocampus, the CCKBR was significantly upregulated in AD-recipient rats vs CT-recipient rats (Fig. 9D). The CCKAR of AD-recipient rats was significantly upregulated in the hippocampus and cortex compared with the control group (Fig. 9C and E). However, there was no significant difference in the expression levels of CCKAR in the frontal lobe between the two groups (Fig. 9B). Taken together, our results showed that AD patients’ gut microbiota could induce the disturbances of CCK receptors (especially CCKAR) in rats, which may be one of the molecular mechanisms involved in the development of alcohol dependence.

## DISCUSSION

AD is a chronic recurrent psychiatric disorder, and long-term heavy alcohol consumption is a sufficient and necessary condition for this disease. However, the patient’s clinical symptoms will persist after alcohol withdrawal, and this disease is extremely prone to relapse. We speculate that other factors besides alcohol promote the development and relapse of AD. As an addictive substance, alcohol is mainly ingested by drinking, and its direct effects on the gastrointestinal tract are obvious. Alcohol abuse induces microbiome dysbiosis, and there was no correlation between the duration of sobriety and the presence of dysbiosis (27). Hence, the effects of alcohol on gut microbiota dysbiosis are not temporary but rather long lasting in AD patients. There is a bidirectional relationship between gut microbiota dysbiosis and alcohol dependence. Alcohol consumption may influence the compositions of gut microbiota and mycobiota and induce dysbiosis and gut barrier impairment. On the other side, gut microbiome dysbiosis may promote the development of alcohol craving and dependence through the “microbiome-gut-brain axis.” However, which kinds of microorganisms play a crucial role in promoting the development of AD? The previous studies on animal models and human patients with AD had revealed inconsistent results in different literature. Wang et al. reported that *Clostridiales* and *Odoribacter* increased in AD mice compared with control mice (28). Another study showed that *Firmicutes* and *Bacteroidetes* reduced and *Proteobacteria* and *Actinobacteria* increased in the chronic alcohol-fed mice (29). Our previous studies have indicated that the commensal microbes *Lachnospiraceae*, *Prevotellaceae*, and *Ruminococcaceae* were dramatically decreased in AD rats (21). In the clinic AD patient studies, Leclercq and colleagues demonstrated that *Lactobacillus* spp. and *Bifidobacterium* spp. were reduced in AD patients, and alcohol abstinence alone resulted in a restoration of suppressed levels of these beneficial bacteria (30). However, some literature showed that AD patients were characterized by the reduction of *Akkermansia muciniphila* and *Faecalibacterium prausnitzii* (14, 15). In this study, we found that *Saccharimonadaceae, Lachnospiraceae*, and *Fusobacterium* increased remarkably in AD patients, and three genera in the family *Ruminococcaceae* decreased in AD patients. Our results are inconsistent with the data of the above documents. Combining the results of existing studies on the gut microbiota of AD patients, there are no consistent results. Hence, it is inappropriate to emphasize that a particular bacterium plays an important role in the development of alcohol dependence. Gut microbiota should be considered as a whole, and maintaining the balance of intestinal homeostasis plays an important role in improving host health (31).

Besides bacteria, fungi are also important members of the commensal microbiome in the human and animal intestines, which have important functions in host health. The changes in gut mycobiota have been associated with disease (32). In recent years, the mycobiome has been an interesting topic in alcohol use disorder patients. However, there have only been a few studies investigating mycobiome composition changes in alcohol-related diseases (2). In this study, we found that the fungal diversity was not significantly altered between AD patients and healthy subjects, and the phylum level of fungi demonstrated no differences between the two groups. However, in the genus level, *Saccharomyces* significantly increased and *Candida* obviously decreased in the AD group compared with the CT group. These results are in conflict with other reports (33, 34). Yang et al. observed that alcohol-dependent patients displayed reduced intestinal fungal diversity and *Candida* overgrowth (33), and Chu et al. also found that fecal levels of *Candida albicans* were higher in AD patients (35). The possible reasons were that the source and time of sampling were different in these studies and all these samples were collected from AD patients with alcoholic hepatitis. Day and Kumamoto well reviewed the changes in the fungal microbiome in the gastrointestinal tract of patients with alcohol-related disease. They reported that the alteration of intestinal mycobiota was not consistent in the different documents (2). Hence, the gut microbiota should be regarded as a whole to comprehensively consider its role in the pathogenesis of the disease. The trans-kingdom network between bacteria and fungi in the present study showed that the ratio of fungi/bacteria, the complexity, and the correlation of the network were significantly reduced in the AD group, which suggests that alcohol consumption alters the bacterial-fungal interactions and disturbs the entire ecosystem balance of the gut microbiota. The balance of gut microbiota may play a key role in modulating the development or pathogenesis of neuropsychiatric conditions (36). Hence, as a whole, the gut homeostasis balance is more appropriate to explain the pathogenesis of alcohol dependence than the alteration of some bacterial genera.

Since most of the intestinal microbiota are non-culturable, the function of the research is limited. At present, FMT is one of the most effective methods to study the function of gut microbiota. FMT has been successfully used to investigate the causal relationship between gut microbiota and psychiatric disorders, such as autism (37, 38), schizophrenia (39), and substance use disorders (40, 41). To reconstruct the phenotype model of gut microbiota and explore the role of gut microbiota in the development of alcohol dependence, we used broad-spectrum antibiotics to deplete the gut microbiota of Wistar rats and then inoculated the fecal microbiota coming from AD patients and healthy subjects in the present study. Our results showed that recipient rats had not obtained 100% gut microbiota from human donors. Approximately 45% of the donor microbiota were successfully colonized in the recipient rat. This result is similar to others’ reports (41). We think this may be related to the intestinal environment of rats, and other kinds of microorganisms are not suitable for colonization in the intestines of rats. However, compared with the CT-recipient rats, the AD-recipient rats showed gut microbiota dysbiosis characteristics, especially the ITS/16S ratio, and the complexity of the microbiome network was significantly reduced, which is similar to the characteristics of gut microbiota in its respective human donor. Hence, our establishing model is suitable to investigate the consequences of gut microbiota on the behavior of rats and the development mechanism of alcohol dependence.

Overall, the results of the FMT experiment in our study revealed that rats transplanted with the feces of AD patients exhibited increased anxiety- and depression-like behaviors and reduced cognition-like behaviors. These behavioral alterations are also the main clinical symptoms of alcohol-dependent patients. Leclercq and colleagues also used the FMT experiment to prove that AD-recipient mice showed reduced sociability and increased depression-like behavior, which was consistent with our results (41). Thus, the gut microbiota may play a role in the development of alcohol addiction. Excluding the alcohol factor, could the gut microbiota induce alcohol preference in rats? Previous literature reported that there was a significant spontaneous alcohol preference in mice transplanted with the feces of AD patients (18). In the present study, our data also revealed that the AD-recipient rats presented a higher alcohol preference ratio than the CT-recipient rats. In addition, some researchers have also demonstrated that FMT with fecal health donors can reduce alcohol-induced depression-like and anxiety-like behaviors in human AD patients and animal models of chronic alcohol exposure (42, 43). Taken together, all these data indicate that gut microbiota plays a potentially vital function in the development of alcohol addiction.

The brain reward circuit was considered to be the main biological basis of substance addiction, which was regulated by various neurotransmitters and their receptor’s expression (44, 45). The gut microbiota plays a pivotal role in the metabolism of neurotransmitters. It not only mediates the functions of the serotonin precursor tryptophan and the serotonergic system but also promotes the synthesis and release of GABA, 5-HT, acetylcholine, norepinephrine, and dopamine (46–49). Although these neurotransmitters primarily act locally in the gut regulating appetite and gastrointestinal function, current evidence confirms that the gut microbiota can communicate with the central nervous system through complex pathways, including the enteric nervous system, endocrine, immune activation, and microbial metabolites, and thereby influencing brain function, mood, and behavior (20, 45, 50). In the present study, we found the levels of BDNF and CCK were significantly altered in AD patients’ peripheral serum. As a neurotrophic factor, the BDNF regulates a variety of neuronal processes and implicates the development of alcohol addiction (51). CCK is the most abundant neuropeptide, widely distributed in the intestine and brain. CCK could interact with multiple neurotransmitters, such as DA, GABA, 5-HT, and endogenous opioids, to modulate the mesolimbic reward system, anxiety, and satiety (52), which are involved in positive and negative reinforcement processes of the development of dependence. Hence, we focused on the changes in BDNF and CCK receptor expression in the rat’s brain after FMT. We found that the expression level of BDNF in the brain showed no obvious changes between the two groups after FMT. However, after receiving AD fecal microbiota transplantation, the level of CCKA receptors was upregulated in the cortex, hippocampus, and frontal lobe, and the level of CCKB receptors also increased in the cortex and frontal lobe in rats. The alteration of CCK and its receptors has been reported in substance addictions such as cocaine, methamphetamine, and alcohol, and the activation of CCK receptors has also been shown to modulate the mesolimbic reward system and anxiety behavior (52). For example, Tracey and colleagues reported that the CCK messenger ribonucleic acid (mRNA) levels throughout the mesolimbic DA pathway increased in rats with methamphetamine administration (53). The levels of ventral midbrain CCK expression had an inverse relationship with DA neuron activity, which is associated with locomotor activity and drug self-administration (54). Hence, blockading or activating CCK and receptor expression regulates the level of DA in the brain, which in turn affects the reward circuit and addictive behavior. Indeed, some researchers have suggested that CCKBR blockade or CCKAR genetic knockout could lead to increased DA levels in the striatum and induce the reaction to cocaine or amphetamine administration (55, 56). CCKAR receptor blockade also decreased behavioral sensitization in amphetamine-administrated rats (57). The CCKBR signal in the brain has anti-reward effects, and CCKBR receptor antagonists may have utility in treating cocaine addiction (58).

In the related research on CCK and AD, Lodge and colleagues reported the CCK mRNA level in the ventral tegmental area was 33% lower in the alcohol-preferring rats compared with non-alcohol-preferring rats (59). The relationship between the CCK receptor and AD has also been shown in animal and human studies. Blockading the CCKA and CCKB receptors could reduce alcohol intake and anxiety-like behavior after ethanol withdrawal (60). The possible mechanisms of CCK and receptors in regulating motivation for alcohol consumption and related emotional behaviors were well reviewed in recent literature (61). However, previous research mainly focused on the role of alcohol-induced changes in CCK and its receptors in alcohol addiction but did not involve the gut microbiota. In the present study, we observed that alcohol-induced changes in gut microbiota caused the alteration of peripheral CCK concentration and CCK receptor expression in the brain, which was consistent with alcohol-induced changes. Therefore, we speculate that the changes of endogenous CCK and CCKR induced by disordered gut microbiota in AD patients may regulate the expression of neurotransmitters such as DA and 5-HT in the specific regions of the brain, which participate in the positive or negative feedback effects on the brain reward circuit. This hypothesis may be considered as a complement to explain the molecular mechanisms underlying the development of alcohol dependence. However, in this study, we did not further deeply investigate the related function of CCK/CCKR changes induced by gut microbiota in the development of alcohol addiction, which is a weakness for our present work. We will use the CCKR gene knockout rats or CCKR agonist/antagonist to verify this hypothesis in the next stage of our study.

### Conclusions

In conclusion, our findings reveal that (i) the intestinal microbiome dysbiosis, not only gut bacteria but also gut fungi, is present in alcohol-dependent patients; (ii) alcohol overconsumption seriously disturbs the gut equilibrium between bacteria and fungi, reduces the interactions among bacterial-fungal trans-kingdom, and increases intestinal permeability. Gut microbiota should be considered as a whole instead of focusing on some species to study the cause of AD; (iii) the rats receiving the microbiota transplantation from AD patients showed some behavioral alterations associated with alcohol dependence, including increased anxiety- and depression-like behaviors, reduced exploratory and recognition memory, and higher alcohol preference; and (iv) the gut microbiota dysbiosis induced by alcohol may involve the development of alcohol addiction through regulating the endogenous CCK and related receptors. Hence, regulating the balance of gut microbiota and the endogenous CCK or CCKR may be a potential strategy for reducing the risk of relapse in alcohol addiction patients.

## MATERIALS AND METHODS

### Participants

We recruited 264 patients from the Department of Addiction Medicine, Henan Mental Hospital, during the inclusion period from January to June 2021. These patients were diagnosed with alcohol dependence according to the Diagnostic and Statistical Manual of Mental Disorders 5 (DSM-5) criteria by a psychiatrist. These patients were admitted to the ward of addiction medicine for detoxification and rehabilitation. They had kept drinking until the day of admission to the detoxification ward. After strict screening by inclusion and exclusion criteria, a total of 34 AD patients were selected for the present study. Any treatments of influence on the gut microbiota were included in the exclusion criteria. including ongoing or recent use of antibiotics, probiotics, or prebiotics (last 3 weeks); a history of inflammatory bowel disease, infectious gastroenteritis, functional digestive disorders, other drug addictions, and cognitive impairment. Nineteen healthy control participants were recruited from the population of physical examination in the second affiliated hospital of Xinxiang Medical University from May to June 2021. These control subjects were the health population attending for a routine physical exam, and they had no history of alcohol consumption and met the exclusion criteria above. Regarding all participants, the blood sample and the fecal sample were collected on the first day of admission in this study. This study was conducted according to the Declaration of Helsinki and was approved by the Human Research Ethics Committee of Xinxiang Medical University (No. XYLL-20210319). None of the participants were paid for their involvement in the study, and all subjects signed the informed consent before inclusion.

### Serological parameter testing

The blood samples were collected from veins and centrifuged in 3,000 rmp/min for 10 min, at 4°C, to collect the serum. Then, ELISA technology was used to measure the serological parameters, including i-FABP, LPS, BDNF, S100B, and CCK, according to the manufacturer’s instructions. The following commercial ELISA kits were obtained from Shanghai Enzyme-Linked Biotechnology Co. Ltd. (Shanghai, China): i-FABP (catalog number 20210923A), LPS (catalog number 20210915A), BDNF (catalog number ML530612-2), S100B (catalog number ML027617-2), and CCK (catalog number ML239315-2).

### Analysis of gut microbiome

The total DNA genes of fecal samples were extracted using Quick-DNA Kit for feces (Qiagen, Germany) following the manufacturer’s instructions. The purity and concentration of genomic DNA were detected by a NanoDrop 2000 spectrophotometer (Thermo Fisher Scientific, USA). For gut bacterial analysis, the universal primer (338F, 5′-ACTCCTACGGGAGGCAGCAG-3; 806R, 5′-GGACTACNNGGGTATCTAAT-3′) was used to amplify V3-V4 hypervariable regions of the 16S rRNA genes by PCR. For gut fungi, the internal transcribed spacer region 1 (ITS1) was amplified with the primer of ITS1F/ITS2R (ITS1F, 5′-CTTGGTCATTTAGAGGAAGTAA-3′; ITS2R, 5′-GCTGCGTTCTTCATCGATGC-3′). After the PCR products were purified, the amplicons were sequenced on the MiSeq PE300 platform (Illumina, San Diego, CA, USA) according to the standard protocols provided by Majorbio Bio-Pharm Technology Co. Ltd. (Shanghai, China). The raw reads were filtered by Divisive Amplicon Denoising Algorithm 2 (DADA2) in Quantitative Insights Into Microbial Ecology (QIIME2 v.2018) quality filters. After removing unqualified base sequences, the amplicon sequence variants (ASVs) were generated as the real sequence information in the sample. Taxonomy was assigned and aligned to the relative database (silva138/16s_bacteria; unite8.0/its_fungi) at 99% sequence similarity, at 70% confidence using the Ribosomal Database Project (RDP) Classifier 2.8. The analysis of bioinformation mainly includes alpha diversity, beta diversity, and microbiome composition differences at various levels of classification. All the bioinformatics data were analyzed on the free online platform of the Majorbio Cloud Platform (www.majorbio.com).

### Fecal metabolome analysis

We selected 24 fecal samples (14 AD group and 10 CT group) for metabolome analysis. The fecal supernatant samples were prepared by dissolving, grinding, ultrasonicating, incubating, and centrifugation according to the previous document (62). A 10µL supernatant from each sample was mixed as quality control (QC) to evaluate the stability during the experiment. Then, the metabolic profiles of all fecal samples were investigated using liquid chromatography and mass spectrometry (LC-MS). Detailed chromatographic and MS conditions were described previously (21). A detailed description of metabolome testing is provided in the supplemental information. The MS raw data were extracted with the softwares of ProteoWizard (v3.0.9134) and the XCMS package, and obtain the characteristic peak information contained the retention time (RT), the mass-to-charge ratio (m/z) value, and peak intensity. According to the peak values of metabolites, the univariate analysis (Student’s *t*-test and fold change analysis) and multivariate analysis (PCA and OPLS-DA analysis) were performed to detect the differentially enriched metabolites between the AD patients and control healthy subjects. For screening the differential metabolites, the variable importance in projection scores ≥ 1.5 and *P* < 0.05 were used in the present study. The databases including the HumanMetabolome Database (HMDB, http://www.hmdb.ca) and the Kyoto Encyclopedia of Genes and Genomes (KEGG, http://www.genome.jp/kegg) were used to identify the metabolites.

### Fecal microbiota transplantation

Six- to eight-week-old, 180- to 200-g Wistar male rats (*n* = 40) were obtained from Charles River Laboratories (Beijing, China; no. 20220310Aazz0619000960). Rats were housed in individually ventilated cages (IVC) with controlled temperature (21 ± 2°C) and humidity (50% ± 5%) in which a 12–12-h light-dark cycle was maintained and were provided autoclaved water and chow *ad libitum*. All animal experimental procedures were approved by the Animal Care and Use Committee of Xinxiang Medical University, China (HMH.No20211111AEC020). All rats were randomly separated into two groups, the CT-recipient group (*n* = 20) and the AD-recipient group (*n* = 20). Each group of rats was housed in six cages with three to four rats in each cage. Fecal samples from AD patients and healthy controls were prepared as previously described (38). Briefly, 34 AD patients and 19 health subjects’ fecal samples were collected in sterile containers. Each fecal sample (1 g) was suspended in 5 mL sterile phosphate buffer solution (PBS), followed by the vortexes for 10 min and sedimentation for 10 min. Then, the slurry was passed through a 100-μm cell strainer. The suspension of the same group was mixed as microbiota donors and immediately administered to the mice by oral gavage. Figure 7A shows the FMT procedure; the gut microbiota from humans were transferred to Wistar rats. Briefly, all rats were treated with a cocktail of antibiotics (0.5 g/L vancomycin, 1 g/L kanamycin, 1 g/L ampicillin, and 1 g/L metronidazole) daily for 2 weeks. Then, 2 mL fecal microbiota from either AD or CT human donors was transferred to microbiota-depleted rats by gavage every day, for 3 weeks. The behavioral tests and alcohol preference tests were performed following the final microbiota transplantation, and at the end of the experiment, all rats were sacrificed, and the colonic contents and brain tissue samples were collected for further analysis.

### Behavioral testing

All behavioral tests were recorded by a video camera connected to a computer, and data analysis was performed by Smart (version 3.0, Panlab, Spain) or Super Maze (version 2.0, Shanghai, China). All rats were transported to the behavioral testing room for a 1-h habituation period. Each apparatus was thoroughly cleaned with water and dried between each animal. Tests were performed with individual rats in random order. Open field test and Elevated plus maze test were used to assess anxiety-like behaviors. A forced swimming test was performed to investigate depression-like behaviors. Y-maze and novel object recognition test (NORT) were used to analyze the cognitive-like behaviors. A detailed description of all these behavioral testing experiments is provided in the supplemental information. To further assess the influence of FMT on the drinking behaviors of rats, we performed an alcohol preference test as described before (63). Briefly, rats were provided with two bottles of drink (4% alcohol and tap water) for free drinking. The positions of the two bottles were exchanged every 12 h to eradicate the effect of position preference. Then, the consumption of liquids was measured every 24 h and for 5 days. The alcohol preference ratio was calculated for analysis of the preferred degree for alcohol.

### Immunofluorescence and Western blotting

The rats were infused with 4% paraformaldehyde through the cardiac artery following anesthesia. The brain tissues (including the frontal lobe, hippocampus, and cortex) were quickly collected after perfusion for fixation, dehydration, and embedding. Frozen brains were sectioned at 10 µm thin slices using a freezing microtome (Leica CM1950). The sections were immersed in sodium citrate buffer for antigen retrieval after equilibration at room temperature for 30 minutes. Then, they were incubated with primary antibodies (including CCKAR, CCKBR, and BDNF) at 37°C for 24 h. After washing with PBS three times, the sections were incubated with fluorescent secondary antibodies labeled with Cy3 (A0521, Beyotime, China) or FITC (A0562, Beyotime, China) at 37°C for 2 h. Then, cell nuclear was stained with DAPI solution (C1005, Beyotime, China), and the results were taken with a fluorescence microscope. Western blotting was used to analyze the expression of CCKR and BDNF in different brain areas. Briefly, the brain tissues were homogenized and the proteins were separated using 10% SDS-PAGE and transferred to a polyvinylidene difluoride (PVDF) membrane. The PVDF membranes were immersed in TBST (Tris-buffered saline with 0.1% Tween-20 and 5% skimmed milk) for 3 h and incubated with primary antibodies including CCKAR, CCKBR, BDNF, and β-actin at 4°C for 24 h. Then, the PVDF membranes were washed with TBST three times and incubated with horseradish peroxidase-conjugated anti-mouse IgG for 2 h at room temperature. At last, the ECL solution was added and the image was exposed to a chemiluminescence imager.

### Statistical analysis

All statistics were performed using SPSS version 22.0, and graphs were made with GraphPad Prism 9 or R package (version 3.6.2). If the data met with normal distribution, unpaired Student’s *t*-tests were used to compare various parameters between AD patients versus CT subjects and AD-recipient versus CT-recipient rats. If the data were not met, non-parametric tests were used to compare the results between groups. *P*-values of <0.05 were set as a threshold for statistical significance. For microbiota analysis, the alpha diversity was assessed according to the Chao, Simpson, and Shannon indices. The beta diversity indices (analyzed using PCoA) were calculated based on unweighted UniFrac distances at the ASV level. A permutational analysis of variance was performed to assess the variation in the taxonomic structure of microbiota communities between groups. LDA and LEfSe analyses were performed to compare biomarkers between groups. For metabolites analysis, after normalizing the raw data, PCA and orthogonal to partial least-squares discriminate analysis were employed to characterize metabolic perturbation and differences among groups. The different metabolites’ pathway topology was analyzed with MetaboAnalyst version 3.0. Correlation analyses were performed using Spearman’s rho correlation test. The correlations among main gut bacterial genera, fungal genera, and fecal metabolites were assessed by linear regression analysis while adjusting for alcohol consumption. The trans-kingdom network figures were built using the package igraph (version 1.2.6). The gray levels of immunofluorescence and WB were assessed using ImageJ software (version 1.8.0).

## Data Availability

The sequencing data of gut microbiota and mycobiota for this study are available in the Sequence Read Archive (SRA) under BioProject ID PRJNA907345. Any requests for data, resources, and reagents should be directed to and will be fulfilled by corresponding author Yan Fan.

## SUPPLEMENTAL MATERIAL

Supplemental material mbio.02392-23-s0001.pdf Fig. S1 to S8; Tables S1 to S6.

ASM does not own the copyrights to Supplemental Material that may be linked to, or accessed through, an article. The authors have granted ASM a non-exclusive, world-wide license to publish the Supplemental Material files. Please contact the corresponding author directly for reuse.
