# Supplementary material for: Intestinal microbiome dysbiosis in alcohol-dependent patients and its effect on rat behaviors
Source: mBio. 2023 Nov 14;14(6):e02392-23. doi: 10.1128/mbio.02392-23 (PMC10746284; doi:10.1128/mbio.02392-23)
Supplement: Supplemental material — Fig. S1 to S8; Tables S1 to S6. [file mbio.02392-23-s0001.pdf]

Supplementary Information

Intestinal Microbiome Dysbiosis in Alcohol-Dependent Patients and its Effect on Behaviors of Rats

Methods

Metabolites Extraction

25 mg of fecal sample was weighed in an EP tube, and 500 μl extract solution (methanol: acetonitrile: water = 2: 2: 1, with the isotopically-labeled internal standard mixture) was added. Then the samples were homogenized at 35 Hz for 4 min and sonicated for 5 min in an ice-water bath. The homogenization and sonication cycle was repeated for 3 times. Then the samples were incubated for 1 h at -40 ℃ and centrifuged at 12000 rpm for 15 min at 4 ℃. The resulting supernatant was transferred to a fresh glass vial for analysis. The quality control (QC) sample was prepared by mixing an equal aliquot of the supernatants from all samples.

LC-MS/MS Analysis

LC-MS/MS analyses were performed using a UHPLC system (Vanquish, Thermo Fisher Scientific) with a UPLC BEH Amide column (2.1 mm × 100 mm, 1.7 μm) coupled to an Orbitrap Exploris 120 mass spectrometer (Orbitrap MS, Thermo). The mobile phase consisted of 25 mmol/L ammonium acetate and 25 ammonia hydroxide in water（pH = 9.75）(A) and acetonitrile (B). The auto-sampler temperature was 4 ℃, and the injection volume was 2 μL. The Orbitrap Exploris 120 mass spectrometer was used to acquire MS/MS spectra on information-dependent acquisition (IDA) mode in the control of the acquisition software (Xcalibur, Thermo). In this mode, the acquisition software continuously evaluates the full scan MS spectrum. The ESI source conditions were set as follows: sheath gas flow rate as 50 Arb, Aux gas flow rate as 15 Arb, capillary temperature 320 ℃, full MS resolution as 60000, MS/MS resolution as 15000 collision energy as 10/30/60 in NCE mode, spray Voltage as 3.8 kV (positive) or -3.4 kV (negative), respectively.

Behavioral testing procedures

Open-field test (OFT):

The OFT was performed from 8:30 a.m. to 12:30 p.m., and the rats were individually tested in an open-field apparatus with 100 cm × 100 cm × 40 cm (length × width × height), version XR-XZ301,

provided by Shanghai Xinruan Information Technology Co.,Ltd. A single rat was gently placed in the corner of the chamber, and after 1 min of adaptation, all spontaneous activities were recorded for 5 min using the video-computerized tracking system. The total motion distance was used as an index of locomotor activity, while time and distance spent in the center (inner 25% of the surface area) were construed as an index of anxiety-like behavior.

Elevated plus maze test (EPM)

The EPM is another apparatus for an assessment of anxiety-like behaviors in rodents. This apparatus is elevated 76 cm from the ground plus a platform with 4 arms (2 open arms and 2 closed arms). The rat was placed in the intersection of the 4 arms, facing an open arm, and was allowed to explore for 5 min. A video tracking system (Ethovision 11.0 XT, Noldus, Information Technology) recorded the exact track of each animal, as well as the total distance traveled and the time spent exploring each of the arms. The time spent and the number of entries into the open arms were used to assess anxiety-like behavior.

Forced swim test (FST):

FST was performed to assess depression-like behavior. Rats were placed in an inescapable transparent Plexiglas tank (20 cm x 100 cm) filled with autoclaved water (30 cm from the bottom) at 23±1°C. The test was performed in two sessions: the first session (pre-test) had a test duration of 15 min (in order to avoid the stress of the rats to affect the results of the experiment). 24 h later, it was then followed by the second session (test) of 5 min. A video camera recorded each mouse for 5 minutes and manual scoring was performed by a trained experimenter who was blinded to the group allocation. Duration of immobility and latency to immobility were used to assess depression-like behavior. Water was renewed between each mouse. Before returning to their home cage, the mice were gently dried with paper towels.

Y-maze:

The Y-maze is an apparatus for assessing spatial recognition memory, which consists of three arms (one start arm and two test arms), each 45 cm in length × 10 cm in width × 29 cm in height. During the training phase, one of the test arms was blocked, and rats were allowed to explore the start arm and open test arm for 10 min. 2 h later, they were placed back in the Y-maze for 5 min, with all three arms open. The time spent in the novel arm was used as a measure of spatial recognition memory.

A preference index, a ratio of the amount of time spent in the novel test arm over the total time spent in the novel and familiar test arms, was used to measure recognition memory.

Novel object recognition

The novel object recognition test was done in the same apparatus as the open field test. Rats were first habituated to the apparatus with three sessions of 5 min each. Then rats were placed back into the arena, for the training session with two identical objects, for a period of 5 min. Objects were black-painted wooden cubes or spheres, approximately 4 cm across and high. 90 min after the training session, mice were placed back into the arena, for the retention session with one familiar object the same as that was used in the training session, and one novel object (same color, different shape). The time spent interacting with the two objects was measured manually, defined as rats touching the object or sniffing the object within a distance of 2 cm. The total time spent interacting with objects was used as a measure of exploratory behavior, and the time spent interacting with the novel object compared to the familiar object was used as a measure of retention memory. A preference index, a ratio of the amount of time spent exploring the novel object over the total time spent exploring both objects, was used to measure recognition memory.

Results

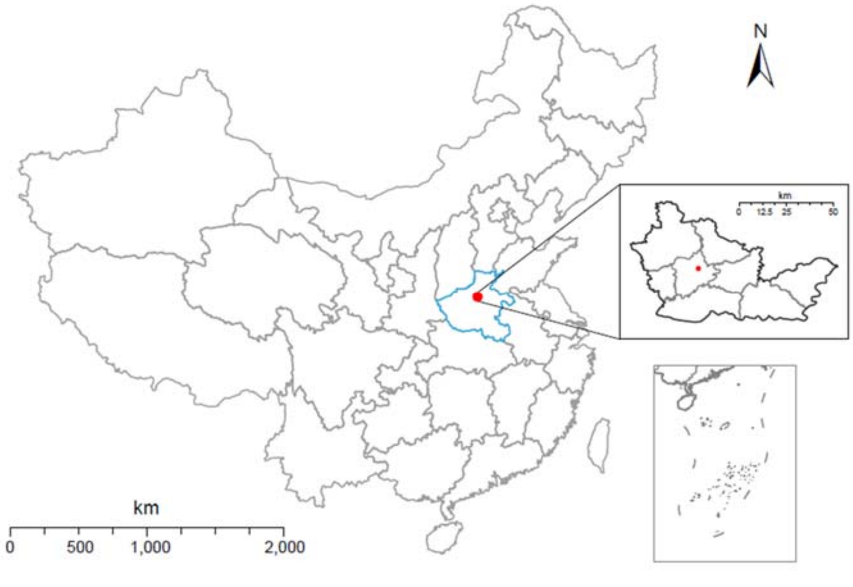

Figure S1. The location of the hospital where the samples were collected. The red dot represents the areas where the samples were collected.

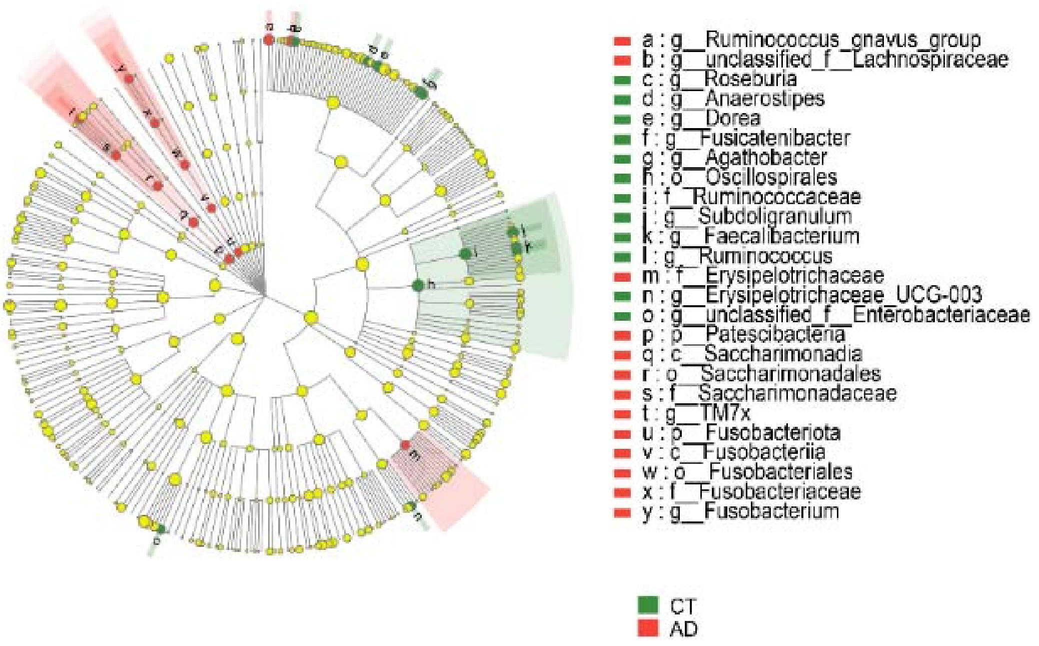

Figure S2. Cladogram of LEfSe Analysis from phylum to genus level between the gut microbiota of AD patients and CT subjects. AD: Alcohol-dependent group; CT: control groups.

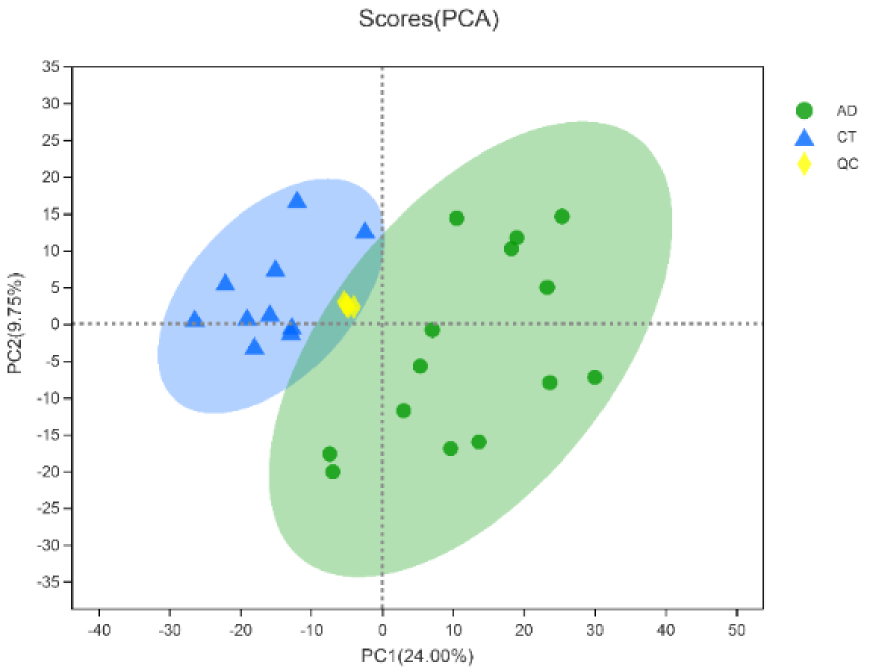

Figure S3. PCA score plots of metabolites in feces from AD vs CT group subjects. AD: alcohol-dependent group; CT: control group, QC: quality control.

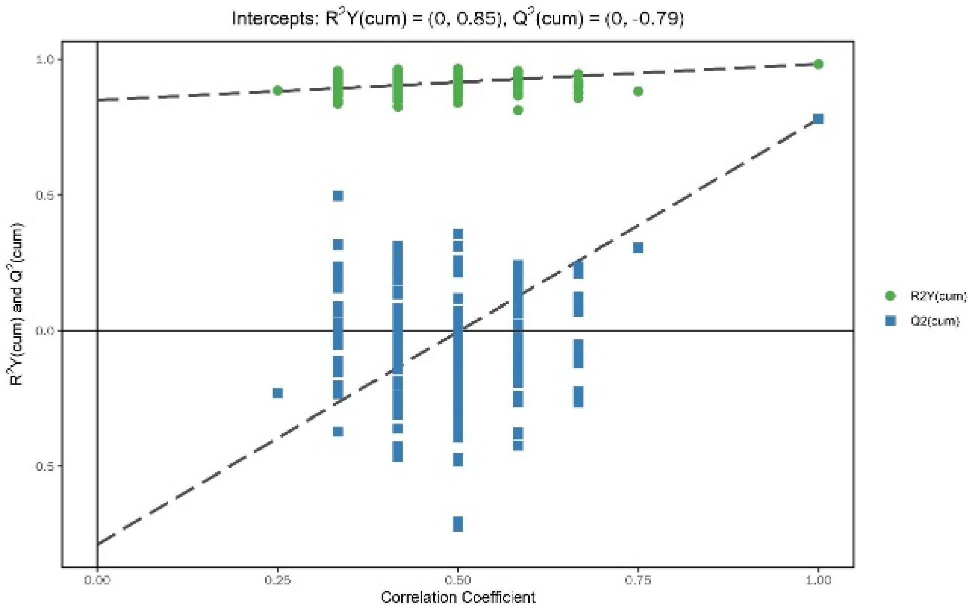

Figure S4. The results of the OPLS-DA model permutation test of the different metabolites between AD and CT groups. OPLS-DA: Orthogonal Projections to Latent Structures-Discriminant Analysis; AD: alcohol-dependent group; CT: control group.

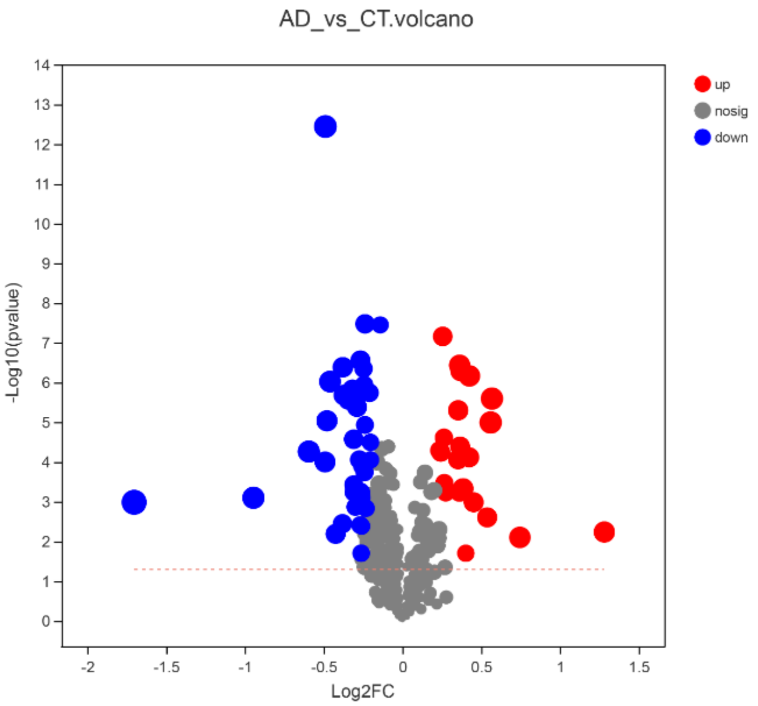

Figure S5. The volcano plot of the differential metabolites between AD- vs CT groups. Red presents the upregulating of metabolites in the AD group vs group. Blue presents the down-regulating in the AD group. AD: alcohol-dependent group; CT: control group.

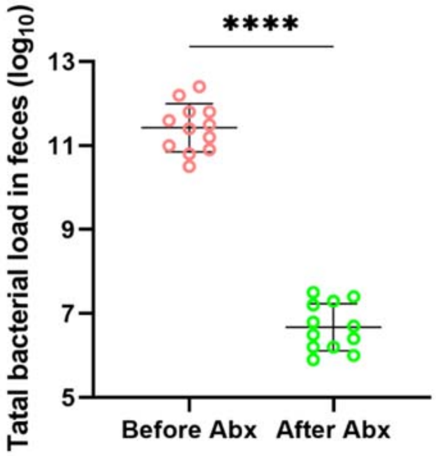

Figure S6. Quantification of the total bacterial load in the feces of rats before and after antibiotics treatment.

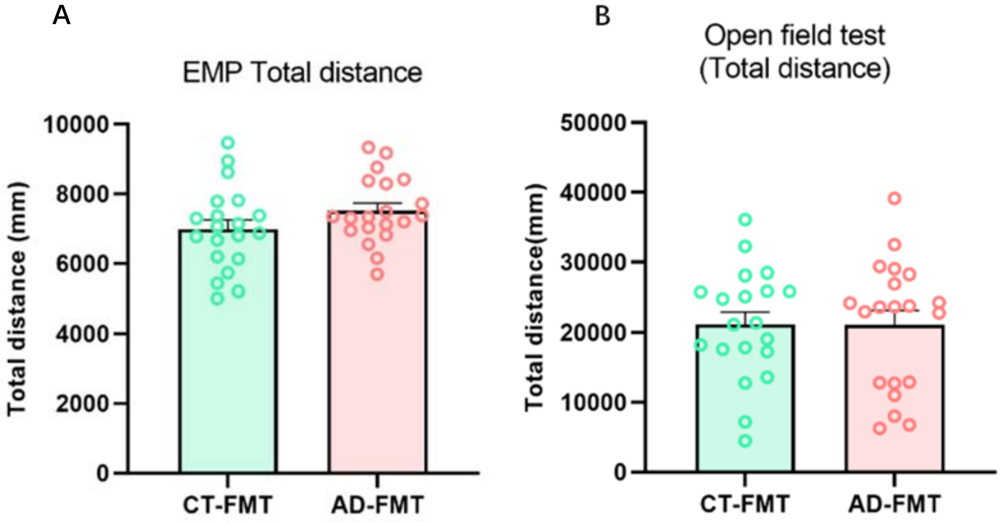

Figure S7. The behavior of rats after FMT. (A) The total distance traveled by rats in the elevated plus maze test, and (B) in the open field test. FMT: feces microbiota transplantation, AD: alcohol-dependent group, CT: control group.

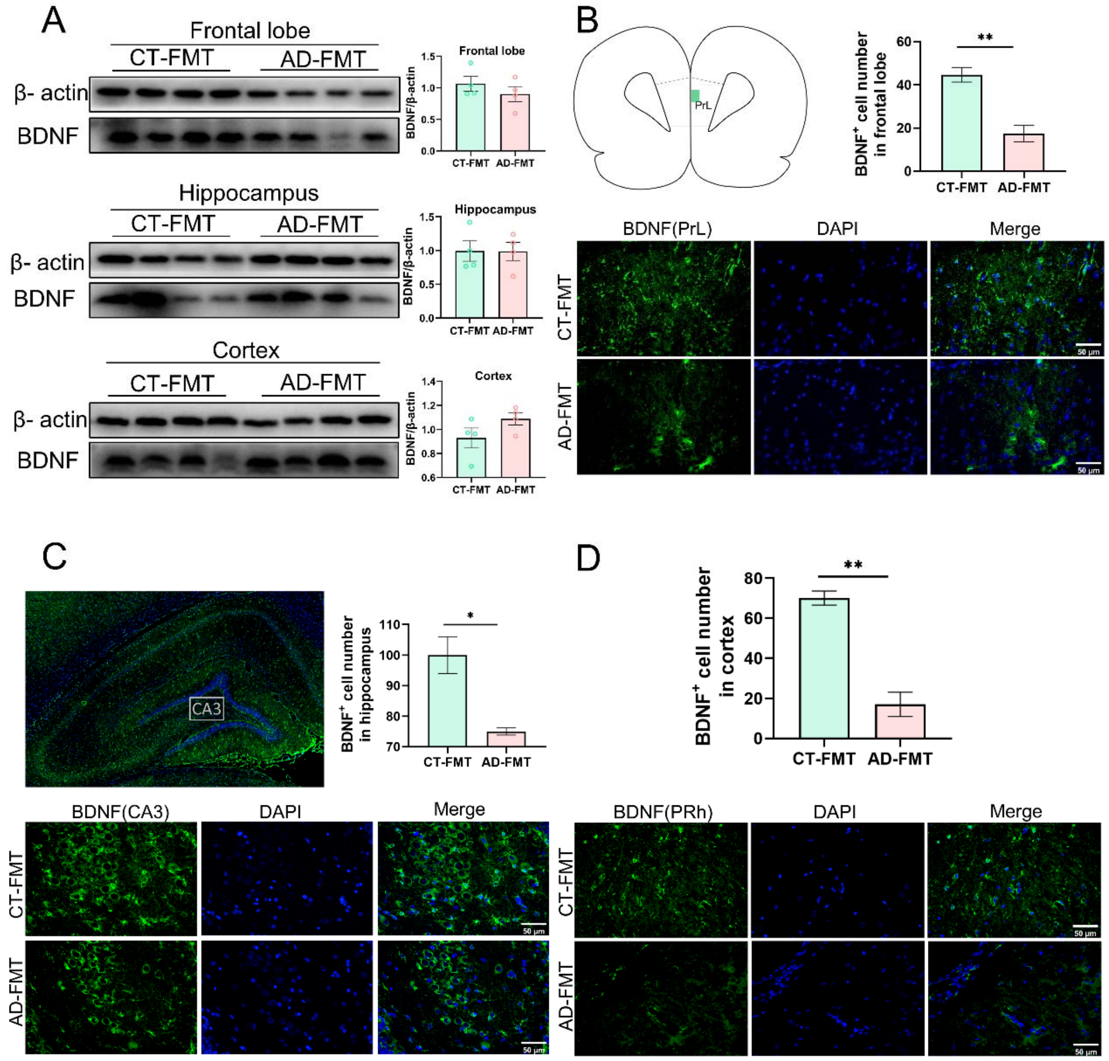

Figure S8. Effect of fecal microbiota transplantation on BDNF expression in the brain. (A) Western blotting images and analysis of BDNF expression in the frontal lobe, hippocampus, and cortex. (B) The expression and analysis of BDNF in the frontal lobe (PrL) were identified by immunofluorescence. (C) The expression and analysis of BDNF in hippocampal (CA3) were identified by immunofluorescence. (D). The expression and analysis of BDNF in the cortex (PRh) were identified by immunofluorescence. Scale bar: 50um in B, C, and D. Data were represented as mean ± SEM. Significant differences were assessed by the Mann-Whitney test or the Unpaired test. *p < 0.05, **p < 0.01.

Table S1. The relative abundance of microbiota in AD and CT groups at phylum levels

Species name	AD median (%)	CT median (%)	P-value
p__Firmicutes	58.58	65.22	0.246
p__Proteobacteria	19.33	19.56	0.829
p__Actinobacteriota	15.49	12.5	0.769
p__Bacteroidota	3.539	2.353	0.156
p__Patescibacteria	1.393	0.1157	0.003
p__Verrucomicrobiota	0.7138	0.1133	0.925
p__Fusobacteriota	0.7694	0.004638	0.002
p__Desulfobacterota	0.1673	0.0558	0.913
p__Cyanobacteria	0.01126	0.07997	0.085
p__Synergistota	0.008733	0.001968	0.417
p__Campilobacterota	0.000938	0	0.214
p__unclassified_k__norank_d__Bacteria	0.000866	0	0.3194
p__Acidobacteriota	0.000577	0	0.497
p__Deferribacterota	0.000361	0	0.319
p__Spirochaetota	0.000144	0	0.497
p__Chloroflexi	7.22E-05	0	0.497

Table S2. The relative abundance of bacteria in genus level

genus name	AD median (%)	CT median (%)	P-value
g__Escherichia-Shigella	13.53	14.19	0.3965
g__Blautia	13.08	12.81	0.7423
g__Bifidobacterium	11.19	8.373	0.6037
g__Romboutsia	5.427	3.24	0.4673
g__Klebsiella	4.876	3.528	0.2514
g__Ruminococcus_gnavus_group	4.324	0.3429	1.37E-06
g__Eubacterium_hallii_group	3.346	4.51	0.09473
g__Bacteroides	3.19	1.864	0.1534
g__Ruminococcus_torques_group	3.142	1.669	0.9517
g__Collinsella	2.937	3.067	0.6587
g__unclassified_f__Lachnospiraceae	2.898	0.5976	0.00104
g__Streptococcus	2.827	1.292	0.3916
g__Megamonas	2.219	0.05411	0.4151
g__Clostridium_sensu_stricto_1	1.877	2.05	0.5107
g__Dorea	1.669	4.202	0.002981
g__Subdoligranulum	1.591	6.912	3.46E-05
g__Lactobacillus	1.234	0.2385	0.2016
g__Faecalibacterium	1.096	6.327	0.000231
g__Ruminococcus	1.015	2.812	0.000315

g__Erysipelotrichaceae_UCG-003	1.004	1.496	0.03793
g__TM7x	0.9988	0.07702	0.01651
g__Agathobacter	0.991	2.526	0.000475
g__Intestinibacter	0.9737	0.5501	0.1934
g__Monoglobus	0.851	0.7088	0.01494
g__Lachnoclostridium	0.8385	0.1436	0.1113
g__Fusicatenibacter	0.8315	3.32	0.000107
g__Butyricicoccus	0.797	0.5138	0.7818
g__Fusobacterium	0.7685	0.004216	0.001135
g__Turicibacter	0.7143	0.2833	0.1594
g__Akkermansia	0.7138	0.1133	0.9245
g__Anaerostipes	0.6798	1.604	0.02987
g__Haemophilus	0.6434	0.05017	0.1051
g__Actinomyces	0.5752	0.06943	4.25E-05
g__Phascolarctobacterium	0.4393	0.03963	0.9929
g__Terrisporobacter	0.3977	0.1363	0.1241
g__Erysipelatoclostridium	0.3252	0.004357	2.00E-07
g__Coprococcus	0.2908	0.7227	0.005119
g__norank_f__Saccharimonadaceae	0.2874	0.03008	0.003934
g__Roseburia	0.2322	0.9284	0.003876
g__Parabacteroides	0.2291	0.08362	0.4839
g__Veillonella	0.2123	0.1879	0.9789
g__Rothia	0.209	0.01152	0.001465
g__norank_f__Eubacterium_coprostanoligenes_group	0.2082	0.5232	0.00277
g__Eggerthella	0.2016	0.03134	0.000943
g__Eisenbergiella	0.1935	0.001687	0.01447
g__Dialister	0.1926	0.06634	0.7501
g__Granulicatella	0.1853	0.05467	0.4306
g__Eubacterium_ventriosum_group	0.1788	0.1282	0.1285
g__Bilophila	0.1605	0.00787	0.3095
g__Clostridium_innocuum_group	0.1497	0.003373	8.74E-05
g__Solobacterium	0.1438	0.01757	0.01381
g__unclassified_f__Enterobacteriaceae	0.1423	1.042	0.02029
g__Weissella	0.1387	0.1671	3.19E-05
g__Gemella	0.1295	0.01251	0.001067
g__Enterococcus	0.1211	0.1887	0.3096
g__UBA1819	0.1039	0.01954	0.02393
g__Atopobium	0.1018	0.008011	0.001147
g__CAG-352	0.09635	0.3783	0.4987
g__Megasphaera	0.08386	0.9522	0.9418
g__norank_f__Lachnospiraceae	0.07744	0.09487	0.1678
g__Ruminococcus_gauvreauii_group	0.06827	0.4378	1.39E-05
g__norank_f__norank_o__Saccharimonadales	0.06596	0.006043	0.001151
g__Faecalitalea	0.06589	0.01335	0.004518

g__norank_f__Oscillospiraceae	0.06134	0.0527	0.5116
g__Tyzzerella	0.05904	0.08714	0.03398
g__Slackia	0.05774	0.09852	0.6316
g__UCG-002	0.05644	0.1217	0.03319
g__Anaeroglobus	0.05593	0.000141	0.2169
g__Flavonifractor	0.05211	0.01181	0.117
g__Hungatella	0.05182	0.000703	0.006036

Table S3. The relative abundance of fungi at the phylum level

Species name	AD Median (%)	CT Median (%)	P-value
p__Ascomycota	73.84	71.25	0.8484
p__Basidiomycota	24.2	27.48	0.6742
p__unclassified_k__Fungi	1.521	0.9632	0.8484
p__Rozellomycota	0.2072	0.2578	0.8062
p__Mortierellomycota	0.1826	0.01575	0.7904
p__Mucoromycota	0.05228	0.006783	0.5955
p__Glomeromycota	0	0.02954	0.1877
p__Chytridiomycota	0.004454	0	0.3113
p__Basidiobolomycota	0.000703	0	0.4945

Table S4. The relative abundance of fungi at the genera level

Species name	AD Median (%)	CT Median (%)	P-value
g__Saccharomyces	38.35	4.594	0.000958
g__Apiotrichum	8.414	5.637	0.8784
g__Candida	8.086	30.95	0.03177
g__Aspergillus	5.394	7.516	0.7988
g__unclassified_f__Aspergillaceae	4.314	3.415	0.8884
g__Tausonia	3.599	0.03282	0.06126
g__Cutaneotrichosporon	3.555	0.1317	0.281
g__Penicillium	3.028	2.347	0.4671
g__unclassified_f__Dipodascaceae	2.514	5.909	0.2252
g__Monascus	2.183	0.08271	0.819
g__Cystofilobasidium	1.609	0.02866	0.1382
g__unclassified_k__Fungi	1.521	0.9632	0.8484
g__Rhodotorula	1.287	0.4438	0.5515
g__Trichosporon	1.235	0.1899	0.1333
g__Cladosporium	1.204	0.9243	0.8083

g__Wallemia	0.8282	1.951	0.6643
g__Talaromyces	0.6559	0.1453	0.6983
g__Debaryomyces	0.6325	0.07243	0.8905
g__Fusarium	0.6284	0.3059	0.2932
g__Cystobasidium	0.5944	0.3258	0.8061
g__Cryptococcus_f__Tremellaceae	0.5917	10.92	0.6097
g__Xeromyces	0.4515	0.9357	0.7899
g__Eurotium	0.4361	0.4302	0.6125
g__Alternaria	0.3313	1.61	0.004057
g__Gibellulopsis	0.294	0.09015	0.2845
g__Bradymyces	0.2809	0	0.4945
g__Thermomyces	0.26	0.1155	0.2034
g__Coprinellus	0.2176	0.5361	0.4357
g__Chaetomium	0.2135	0.01072	0.6064
g__Auricularia	0.2124	1.63	0.2381
g__Saccharomycopsis	0.2021	0	0.1358
g__Mortierella	0.1826	0.001969	0.2667
g__Didymella	0.1586	0.2549	0.5713
g__Coniochaeta	0.1574	0.01663	0.931
g__unclassified_p__Rozellomycota	0.1541	0.2534	0.6279
g__Occultifur	0.153	0.5256	0.01129
g__Filobasidium	0.1515	0.2737	0.1267
g__Exophiala	0.1448	0.06915	0.8738
g__Wickerhamomyces	0.141	0.5702	0.3265
g__Cordyceps	0.1403	0.006127	0.478
g__Diutina	0.1396	0	0.05983
g__Saitozyma	0.1381	0.03414	0.7881
g__Erythrobasidium	0.1362	0.01422	0.6674
g__unclassified_c__Cystobasidiomycetes	0.1323	0.04376	0.273
g__Thelonectria	0.1293	0	0.4945
g__Botrytis	0.125	0.003939	0.1842
g__Issatchenkia	0.1217	0.06039	0.095
g__unclassified_f__Pleurotheciaceae	0.1179	0.01269	0.701
g__Gibberella	0.1124	0.02166	0.3333
g__Trichoderma	0.1093	4.496	0.8928
g__unclassified_p__Basidiomycota	0.1085	0.2901	0.2633
g__unclassified_o__Saccharomycetales	0.1074	0.05864	0.6382
g__Kazachstania	0.103	0.2114	0.3782

Table S5. Differentially Expressed Metabolites profile between AD and CT group

Metabolite name	rt	Mz	VIP	P-value	Q-value	Fold change	Changes (AD vs CT)
Oxazepam	42.1762	287.0570938	2.389315518	8.1445E-05	0.025965387	36.62668168	↑
Carbendazim	318.652	192.0765354	2.354394189	0.006890474	0.054739905	0.065772746	↓
(2S,2'S)-Pyrosaccharopine	481.988	259.1286652	2.322753329	0.01813871	0.075203814	0.130442265	↓
Harman	41.1072	183.0911245	2.282811606	0.022274723	0.078740642	0.108647763	↓
3-Furanmethanol glucoside	318.006	261.098193	2.260321044	0.004026014	0.045748347	0.106522281	↓
Loquatoside	357.233	439.1197886	2.180210752	0.016185039	0.073180867	0.015008656	↓
Proline betaine	216.249	144.1014785	2.167377671	0.001411584	0.034889708	33.14907763	↑
2-Aminonaphthalene	214.525	144.0805501	2.145198621	0.001865921	0.03638046	29.24455814	↑
L-Octanoylcarnitine	223.805	288.2167191	2.13908509	0.034937137	0.089190944	26698.19924	↑
Alantolactone	389.919	255.1338112	2.129495446	0.002354347	0.038033994	0.220965344	↓
Acetylvalerenolic acid	404.7525	315.1540981	2.118287078	0.028697418	0.08449806	0.127226298	↓
Kaempferol 3-glucuronide	276.9075	463.0902236	2.085479263	0.012496871	0.068157201	9563.128589	↑
PE(16:0/14:0)	166.312	664.4907835	2.065079141	2.83062E-05	0.024750215	6.137577142	↑
2-Phenylethanol	187.18	105.069544	2.003320287	0.029822824	0.085389676	6.453131982	↑
1H-Indole-3-acetamide	74.623	175.0862838	1.982002973	0.000148375	0.026275371	5.422292423	↑
(2R,3R,4R)-2-Amino-4-hydroxy-3-methylpentanoic acid	266.241	148.0964424	1.980160738	0.005967209	0.051821555	0.332615768	↓
Valenciachrome	197.1925	413.3012466	1.976397148	0.016094545	0.073078248	9.659441942	↑
Nerolidol3-O-[a-L-rhamnopyranosyl-(1->4)-a-L-rhamnopyranosyl-(1->6)-b-D-glucopyranoside]	229.791	677.374374	1.969486451	0.010012739	0.063417437	9.279279434	↑

Capsiamide	38.8193	270.2787837	1.953767935	0.001580741	0.035442649	10.11829597	↑
Queretaroic acid	244.2365	473.359067	1.937329651	0.001082377	0.033408328	28.5900624	↑
2,6-Dimethylpyrazine	46.61295	109.075684	1.934552471	0.028046985	0.083959297	0.199151278	
Betaine	285.2815	118.0858303	1.924022561	0.000421961	0.029905704	3.203958026	↑
2-Methylbutyroylcarnitine	259.5085	246.1691036	1.922426748	0.019118008	0.076096474	117.9162568	↑
Pandamarilactam 3x	372.769	236.1275062	1.908080318	0.011546347	0.066507834	10.7975536	↑
4,8-Dimethylnonanoyl carnitine	205.84	330.2630392	1.907804624	0.007535289	0.056875562	10.62639814	↑
Methylimidazole acetaldehyde	91.6097	125.0705928	1.895887784	0.03756001	0.091087	0.193304481	↓
Biliverdin	252.662	583.2544702	1.878436412	0.005578981	0.050683437	4.176007746	↑
L-Carnitine	367.412	162.1120919	1.877485785	0.000170676	0.02632534	3.785027327	↑
N, N-Dimethylaniline	187.238	122.0960173	1.85098937	0.029550438	0.085178417	5.957950095	↑
3-Methylcyclohexanethiol	321.817	131.0892268	1.850601567	0.004132752	0.046154763	0.317656709	↓
Beta-Carboline	43.7671	169.075561	1.844836087	0.005641583	0.050844673	0.233564065	↓
2-Pyrrolidineacetic acid	321.726	130.0858913	1.833092086	0.004907549	0.048776309	0.333353135	↓
Furcelleran	250.129	478.1966682	1.826132991	0.011632834	0.066665526	3.312108681	↑
2,6-Dimethylheptanoyl carnitine	215.972	302.2317961	1.813036833	0.007071858	0.055363	6.210462768	↑
25-Hydroxyvitamin D2	31.2628	413.3399021	1.809807468	0.000520253	0.03063269	0.395283421	↓
Tryptamine	214.237	161.1070443	1.795929042	0.002237272	0.037512299	22.28419602	↑
Mangiferdesmethylursanone	29.1062	429.3719345	1.792512348	0.002654149	0.039678005	0.435764298	↓
Flazine	205.0985	309.0865837	1.791466972	0.004565919	0.047670158	0.213417234	↓
Propionylcarnitine	295.995	218.1380149	1.759936691	0.013129309	0.069160806	16.97520251	↑
Pyrimidine	320.501	81.04457199	1.759722357	0.00167894	0.035718939	3.357726027	↑
Creatine	362.661	132.0763278	1.758173473	0.000108966	0.026138042	7.416446847	↑

Dethiobiotin	194.024	215.1384492	1.754047947	0.018159068	0.075223127	0.234908172	↓
Bilirubin	250.333	585.270442	1.74657428	0.000575051	0.030940376	4.785198546	↑
Camelledionol	216.669	441.34011	1.741511056	0.023363495	0.079639153	2.664237549	↑
13-L-Hydroperoxylinoleic acid	199.6235	295.2259609	1.731228004	0.00914869	0.06138909	0.425766203	↓
Tectorigenin 7-sulfate	24.9753	381.0268333	1.706515137	0.023122353	0.079445728	9.920679688	↑
Linoelaidyl carnitine	194.179	424.342241	1.704921529	0.005286043	0.049893267	4.047478746	↑
[6]-Gingerdiol 3,5-diacetate	316.3525	398.2527611	1.699731601	0.00753384	0.056871003	40.06878137	↑
Tetracosahexaenoic acid	273.1265	357.2749576	1.690021609	0.023303492	0.07959131	7.632151553	↑
Prostaglandin E2	412.486	375.2115342	1.68747389	0.00677045	0.054317374	0.310578149	↓
3-Isovalidene-3alpha,4-dihydrophthalide	30.44795	205.1214594	1.684790375	0.046669024	0.096470255	0.35963475	↓
alpha-Micropteroxanthin B	198.262	397.3139665	1.683543377	0.000861743	0.031954155	12.49584446	↑
2-Angeloyl-9-(3-methyl-2E-pentenoyl)-2b,9a-dihydroxy-4Z,10(14)-oplopadien-3-one	196.934	429.2597038	1.68236517	0.004155132	0.046238214	4.419881018	↑
1,2,4-Tris(methylene)cyclohexane	30.5688	121.1007168	1.679914507	0.017097973	0.074169926	0.393347681	↓
p-Mentha-1,3,8-triene	29.91145	135.1163513	1.676910539	0.014769163	0.071469706	0.465247365	↓
Boviquinone 4	96.3029	413.2660709	1.673886083	0.010851482	0.065179922	0.341828595	↓
[12]-Gingerol	197.881	396.3107732	1.644651994	0.000651962	0.031292411	11.0397501	↑
2-Methyl-5-propylpyrazine	219.435	137.107031	1.643221614	0.004956371	0.048926993	2.802018648	↑
Dodecanoylcarnitine	205.103	344.2791667	1.639543529	0.033417593	0.087998178	25.59978557	↑
L-Phenylalanine	273.6325	166.0856441	1.636391781	0.006654041	0.053899522	3.806035755	↑
(3R, 6'Z)-3,4-Dihydro-8-hydroxy-3-(6-pentadecenyl)-1H-	231.0645	373.2733996	1.635801709	0.00074978	0.031643604	15.80068113	↑

2-benzopyran-1-one							
(3beta,17alpha,23S,24S)-17,23-Epoxy-3,24,29-trihydroxy-27-norlanost-8-en-15-one	34.5621	475.3408823	1.635154666	0.010451975	0.064363702	0.413022741	↓
(3beta,5beta,8beta,22E,24xi)-Ergosta-6,22-diene-3,5,8-triol	29.3582	431.3508592	1.630330165	0.017074811	0.074145827	0.416675962	↓
Olomoucine	447.879	299.1593612	1.62704569	0.045788682	0.095998465	0.320620406	↓
Asparaginyl-Hydroxyproline	360.7095	246.1077034	1.625343141	0.041756682	0.093755179	0.262101127	
(2R,6x)-7-Methyl-3-methylene-1,2,6,7-octanetetrol 2-glucoside	429.362	367.1966137	1.623943244	0.007899698	0.057990831	107.5503525	↑
Linoleoyl ethanolamide	32.876	324.2885625	1.622536923	0.004838134	0.048558486	4.433264776	↑
Epidermin	408.952	262.1281996	1.616959986	0.030436783	0.085855664	4.879651754	↑
Tetrahydrofolic acid	432.8675	446.1767976	1.612872834	0.002034116	0.036935632	6.048704725	↑
Tsugaric acid A	238.289	499.3747758	1.59844849	0.009329742	0.061833833	5.844148241	↑
trans-S-(1-Propenyl)-L-cysteine	289.768	162.0578235	1.592563252	0.03587026	0.08988797	0.166554077	↓
3-Methyldioxyindole	212.686	164.0702173	1.582333389	0.00488603	0.048709237	2.640111913	↑
N-Methyl-a-aminoisobutyric acid	313.1985	118.085832	1.578353867	0.003620816	0.044067031	3.1668609	↑
Butyrylcarnitine	275.27	232.1540888	1.56126244	0.008892272	0.060739921	20.56855321	↑
Laccarin	325.9715	195.1123378	1.558095911	0.041189628	0.093418011	0.299011241	↓
7alpha-Hydroxy-3-oxo-5beta-cholan-24-oic acid	257.0865	391.2867039	1.554706223	0.042712026	0.094308196	0.284041623	↓
2,3-Diethyl-5-methylpyrazine	206.743	151.1227337	1.554407628	0.003541667	0.04371943	0.371095584	↓

4-(Glutamylamino) butanoate	314.418	233.1126389	1.551701275	0.012947013	0.068878626	0.293501409	↓
1-Butanethiol	359.2505	91.05812223	1.546757092	0.006964849	0.054997615	3.595469482	↑
Methyl 5-hydroxyoxindole-3-acetate	234.307	222.075771	1.541105557	0.014389393	0.070969062	0.273335585	↓
Alanylglycine	316.5415	147.0840432	1.53622504	0.001114255	0.033583249	3.659091941	↑
2-Methylguanosine	204.466	298.1138337	1.533647759	0.020888324	0.077541308	4.86920688	↑
L-Hexanoylcarnitine	324.2275	260.1849802	1.531190381	0.019658505	0.076559258	0.400957652	↓
Serotonin	128.441	177.1016877	1.530422403	0.003523365	0.043646621	3.746786851	↑
5-Methoxytryptophan	64.3298	235.1070967	1.530375338	0.007733792	0.05749076	0.304004551	↓
Glycerophosphocholine	401.7605	258.1090991	1.524832055	0.013379461	0.069539098	7.934994607	↑
beta-Thujaplicin	30.8791	165.0904103	1.52419358	0.01467298	0.071344702	0.437145023	↓
1-Hexanethiol	313.515	119.0892968	1.514230445	0.005045077	0.049195601	2.958530939	↑
Norophthalmic acid	389.831	276.1186187	1.505008745	0.031492434	0.086625394	0.24703524	↓
Ureidopropionic acid	364.857	133.060575	1.500733424	0.000800388	0.031793977	8.415286282	↑

Table S6. Metabolism pathway enrichment analysis

Pathway	Total	Hits	Raw p	-ln(p)	Impact value
Glycine, serine, and threonine metabolism	48	4	0.0062494	5.0753	0.21239
Vitamin B6 metabolism	32	2	0.089517	2.4133	0.03905
Nitrogen metabolism	39	2	0.12496	2.0797	0
Tryptophan metabolism	79	3	0.12664	2.0664	0.06471
Histidine metabolism	44	2	0.15204	1.8836	0.0247
Biotin metabolism	11	1	0.16089	1.827	0.19512
Lysine degradation	47	2	0.16881	1.779	0.01009
Primary bile acid biosynthesis	47	2	0.16881	1.779	0.06346
Linoleic acid metabolism	15	1	0.2129	1.5469	0
Porphyrin and chlorophyll metabolism	104	3	0.22452	1.4938	0.02945
Cyanoamino acid metabolism	16	1	0.2254	1.4899	0
Thiamine metabolism	24	1	0.31871	1.1435	0
Aminoacyl-tRNA biosynthesis	75	2	0.33308	1.0994	0
Arginine and proline metabolism	77	2	0.34479	1.0648	0.04203
Phenylalanine, tyrosine, and tryptophan biosynthesis	27	1	0.3508	1.0475	0.00062
Methane metabolism	34	1	0.42006	0.86737	0
Purine metabolism	92	2	0.43026	0.84336	0.00878
Glutathione metabolism	38	1	0.45634	0.78451	0
Phenylalanine metabolism	45	1	0.5146	0.66437	0.11906
Steroid hormone biosynthesis	99	1	0.79984	0.22334	0.00391
